# Cytochrome P450 Enzymes Are Associated with the Innate Metabolic Resistance of *Echinochloa crus-galli* to Florpyrauxifen-Benzyl

**DOI:** 10.1021/acs.jafc.6c06421

**Published:** 2026-10-23

**Authors:** Luan Cutti, Eduardo C. Rudell, Crystal Sparks, Guilherme Menegol Turra, Paula Sinigaglia Angonese, Estéfani Sulzbach, Angela Da Cas Bundt, Catarine Markus, Franck E. Dayan, Todd A. Gaines, Aldo Merotto

**Affiliations:** † Department of Crop Science, 28124 Federal University of Rio Grande do Sul, Porto Alegre, Rio Grande do Sul 91540-000, Brazil; ‡ Department of Agricultural Biology, 3447 Colorado State University, Fort Collins, Colorado 80523, United States; § 501878 Corteva Agriscience, Barueri, São Paulo 06460-000, Brazil

**Keywords:** nontarget site resistance, *CYP*, barnyardgrass, temperature, auxin, arylpicolinates

## Abstract

This study aims to identify the resistance mechanism and temperature effects on florpyrauxifen-benzyl (FPB) resistance in *Echinochloa crus-galli* biotypes with no prior FPB exposure. The SAOJER-R biotype was resistant to FPB, with a resistance factor (RF) of 3.80, decreasing to 1.49 when a CYP450 inhibitor was present. Its RF increased from 2.37 at 25/20 °C to 5.22 at 35/30 °C. FPB and florpyrauxifen acid (FPA) levels were lower in the resistant biotype and in rice than in the susceptible biotype. CYP450 inhibition increased the FPA amount recovered, confirming metabolic involvement. Genes from *CYP*, *GST*, *UGT*, *ATP*-binding cassette, *AKR*, and *PER* families were upregulated in the resistant biotype. *CYP71Z*-like, *CYP704C*-like, and *CYP81A*-like genes are putatively involved in metabolizing FPB in SAOJER-R, requiring functional validation. Overall, the results highlight temperature-dependent metabolic resistance and identify new candidate genes involved in FPB resistance.

## Graphical abstract

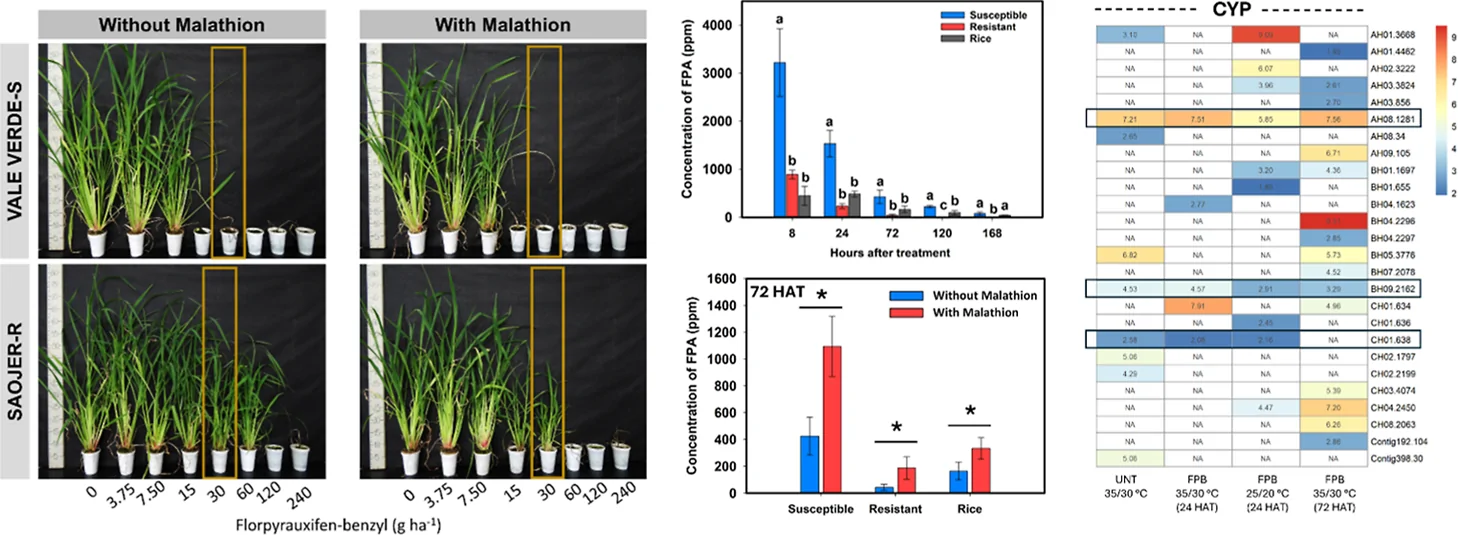

## Introduction

The *Echinochloa* genus is one of the most important herbicide-resistant weeds because of the worldwide occurrence of resistance at high frequency to multiple herbicide modes of action. *Echinochloa* species have been reported to be resistant to quinclorac,^1^ glyphosate,^2^ cyhalofop-butyl,^3^ fenoxaprop-*p*-ethyl,^4^ clomazone,^5^ and all chemical groups of the ALS (acetolactate synthase)-inhibiting herbicides.^6^ Both target site mutations and nontarget site resistance related to enhanced metabolism are well-characterized for ALS inhibitors,^7,8^ ACCase inhibitors,^3,9^ and glyphosate,^10^ while the quinclorac mechanism of resistance is still unclear.^1^ In *Echinochloa* spp. and in other weed species, the most important gene families involved in herbicide metabolism are *CYP* (cytochrome P450 monooxygenase), *GST* (glutathione-*S*-transferase), *UGT* (UDP (UridineDiPhosphate)-glycosyltransferase), *ATP*-binding cassette (*ABC*), and *AKR* (aldo-keto reductase) due to overexpression of specific genes.^12^

*Echinochloa crus-galli* is an allohexaploid species (2*n* = 6*x* = 54) and has 104,780 genes, including 867 *CYP*s, 227 *GST*s, 113 *AKR*s, and 361 *ABC*,^13^ which is higher than diploid grasses like *Brachypodium distachyon*, *Oryza sativa*, *Sorghum bicolor*, *Setaria italica*, or *Zea mays*.^14^ The higher abundance of these metabolic genes could facilitate herbicide resistance evolution, producing a higher number of these promiscuous metabolic enzymes that degrade diverse xenobiotic chemical classes. Enhanced herbicide metabolism is considered the most problematic mechanism of resistance, especially because of its potential to metabolize different herbicides used in the resistance management program, or even new herbicides that have not yet been discovered or brought to the market.^15,16^ Several metabolism genes conferring herbicide resistance have been well-characterized in *Echinochloa*, such as *CYP81A12* and *CYP81A21* conferring resistance to bensulfuron-methyl, penoxsulam, diclofop-methyl, tralkoxydim, and pinoxaden^7,17^ and *CYP709C69* to diclofop-methyl^18^ in *Echinochloa phyllopogon*; *CYP81A68* to penoxsulam, cyhalofop, and metamifop^19^ in *E. crus-galli*; and *AKR4* and *ABCC8* to glyphosate in *E. colona* ^11,20^ .

Florpyrauxifen-benzyl (FPB, chemical name IUPAC: benzyl 4-amino-3-chloro-6-(4-chloro-2-fluoro-3-methoxyphenyl)-5-fluoropyridine-2-carboxylate) is a relatively new rice-selective herbicide.^21^ This compound was considered the solution to manage *Echinochloa* spp. resistance in rice fields due to its high efficiency in controlling populations resistant to other herbicides.^22^ The herbicides FPB and halauxifen-methyl are the newest members of the auxin mimic mode of action, chemical group arylpicolinates. The arylpicolinates are proherbicides, formulated as esters, that are bioactivated in plants into the active compound florpyrauxifen acid (FPA). The arylpicolinates bind to auxin signaling F-box (AFB) proteins with higher binding preference for the AFB5-AUX/IAA coreceptor, like picloram, fluoroxypyr, and quinclorac.^23,24^ Once the synthetic auxin binds to TIR1/AFB coreceptors, it starts a cascade of events by degradation of AUX/IAA repressors^25^ and consequent activation of transcription factors and expression of auxin-responsive factor (ARF) genes, leading to biochemical and physiological events related to the action of auxin herbicides.^26^

FPB resistance in *E. crus-galli* was quickly reported a few crop seasons after its market release. Recent studies of different biotypes have identified increased metabolism as the mechanism of FPB resistance. First, the resistance was associated with decreased translocation and metabolism in biotypes from the midsouthern USA, but the genes involved were not identified.^27^ More recently, a biotype from China had several *CYP* and *GST* genes upregulated in the resistant biotype.^29^ Another study from Hunan province in China, using a different biotype, identified the gene *CYP72A385*, upregulated by the methyltransferase-like Ec*METTL7A*, as the main gene associated with FPB resistance.^30^ This epigenetic regulation indicates that environmental variability may affect the occurrence of resistance to the FPB. Another study indicated that the low expression of the *AFB4* receptor is associated with FPB resistance in a biotype sampled in 2012 in Anhui Province of China.^31^ Variable control of barnyard grass with FPB under field conditions has been observed, but the causes of this are unknown. The objective of this study was to characterize the mechanism of resistance and evaluate how temperature modulates FPB resistance in *E. crus-galli* biotypes that had no prior history of FPB application.

## Materials and Methods

### Screening of *E. crus-galli* Biotypes with FPB and Resistance Confirmation

A total of 20 *E. crus-galli* biotypes (collected before 2019) from South Brazil were first screened with FPB (Loyant, EC, 25 g L^–1^, Corteva Agriscience, Indianapolis, IN, USA) under greenhouse conditions (28 ± 5 °C). These biotypes had never received any FPB application before but had a different resistance background to other herbicide modes of action (Table 1). The initial biotypes (G0—generation 0) have been self-pollinated for at least three generations for use in previous studies. About 50 plants of each biotype were planted in single trays containing soil and commercial substrate, fertilized with N–P–K, and kept flooded. The herbicide FPB was applied at 25 g a.i. ha^–1^ (intermediate label rate) to plants at the three-to four-leaf stage. Survivor plants produced seeds (G1), which were germinated and screened with FPB at 30 g a.i. ha^–1^ (highest label rate). The biotype with the most surviving plants (SAOJER-01) was selected to produce G2 seeds, and the plants were screened at 60 g a.i. ha^–1^ (twice the label rate) and produced G3 seeds. One more selection was performed at 60 g a.i. ha^–1^ on SAOJER-01 to collect the G4 seeds, which were utilized to perform the following experiments. Herbicide treatments were applied using a Greenhouse Spray Chamber Generation III (DeVries Manufacturing) and a TJ8002E nozzle at 200 L ha^–1^ spray volume. The survival evaluation, as the number of plants, was performed at 28 days after treatment (DAT). SAOJER-01 (G0) was collected in a rice field with suspected cyhalofop-butyl resistance and confirmed as metabolic resistance in our previous study.^3^

The biotype SAOJER-01 (from G4), now called biotype SAOJER-R, and the susceptible VALE VERDE-01, now called biotype VALE VERDE-S, were utilized to perform a survival dose–response curve, under greenhouse conditions (28 ± 5 °C). The biotypes SAOJER-R and VALE VERDE-S were collected in the Sao Jeronimo and Vale Verde municipalities, respectively, in Rio Grande do Sul State, Brazil. The doses were 0, 3.75, 7.5, 15, 30, 60, 120, and 240 g of FPB ha^–1^. Each experimental unit was a tray with 50 plants, and each treatment had three replicates. The number of surviving plants and the fresh biomass were evaluated at 35 DAT. The application conditions and plant growth were the same as described before. The data were submitted to analysis of variance (ANOVA), assumptions were checked, and analyzed by a nonlinear three-parameter log–logistic equation using the *drc* package from R software, considering the lack-of-fit test and significance of parameters.^32^

**Table 1:**
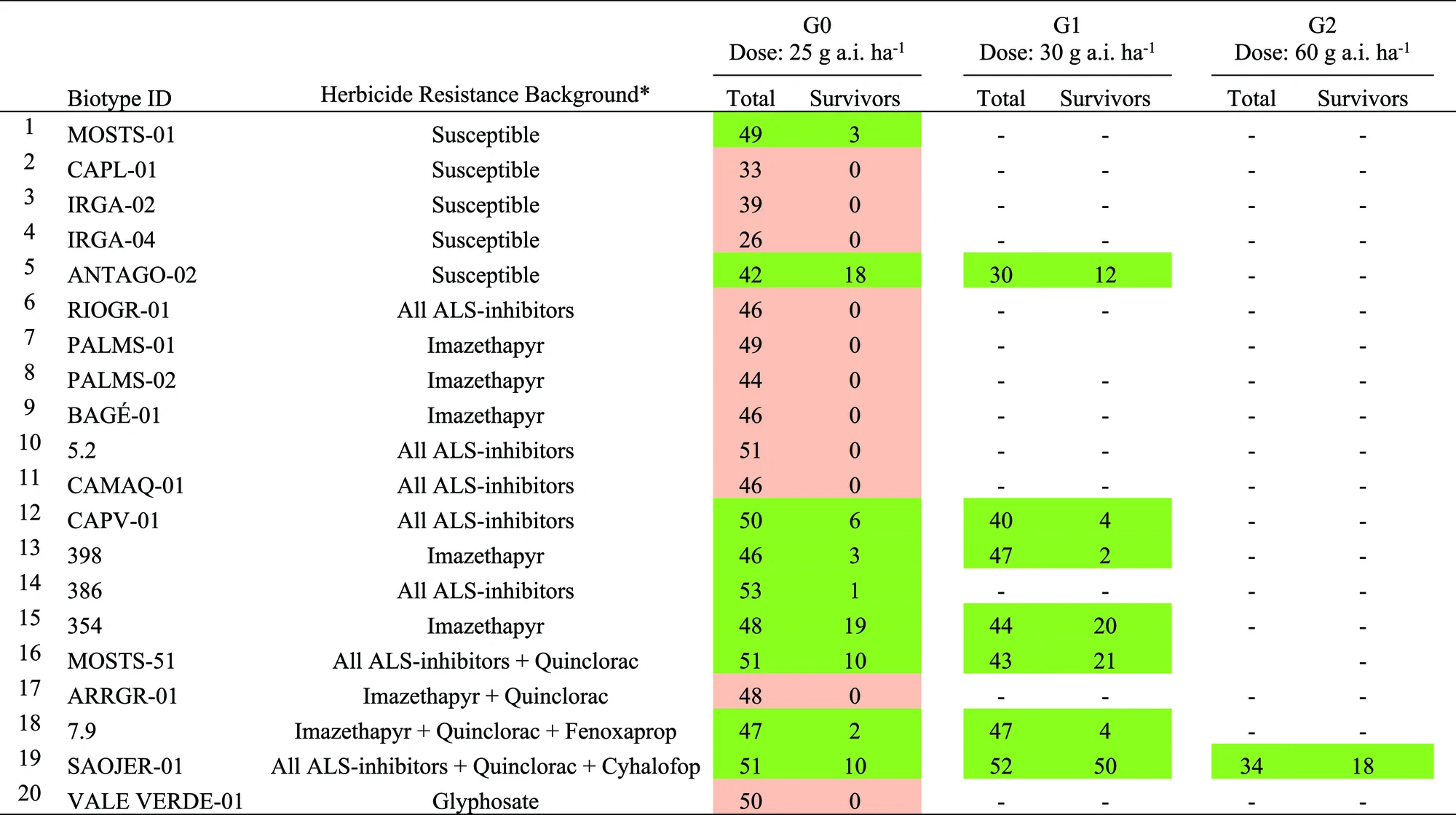
FPB Survival in Different *E. crus-galli* Biotypes^a^ ^,^ ^b^

a Total number of plants evaluated and number of survivors at 28 days after FPB application in *E. crus-galli* biotypes, different generations, and herbicide rates (greenhouse at 28 ± 5 °C).

b Color brown means susceptible; color green means at least one plant survived. *Herbicide resistance previously characterized.

**Table 2:** FPB Survival Dose–Response Curve^a^ ^,^ ^b^

				*e*(ED_50_)		
biotypes	*b*	*d*	*e*(ED_50_)	lower limit	upper limit	RF
	number of surviving plants					
VALE VERDE-S	7.80*	49.84*	10.19*	9.43	10.96	
SAOJER-R	3.15*	49.99*	48.31*	45.51	51.11	4.7*
	fresh biomass (g)					
VALE VERDE-S	5.81*	380.62*	9.97*	8.37	11.57	
SAOJER-R	1.68*	349.41*	44.68*	30.11	59.24	4.5*

a Three-parameter log-logistic model for the survival dose–response curve to FPB with two *E. crus-galli* biotypes, evaluating the number of surviving plants and the fresh biomass (greenhouse at 28 ± 5 °C).

b *b*: Curve slope at the inflection point; *d*: upper limit; *e*(ED_50)_: effective dose to reduce 50% of the variable. RF: resistance factor based on ED_50._ Asterisk (*) or ^ns^ indicates the parameter significance fitting the log–logistic model (*p* ≤ 0.05).

**Figure 1:**
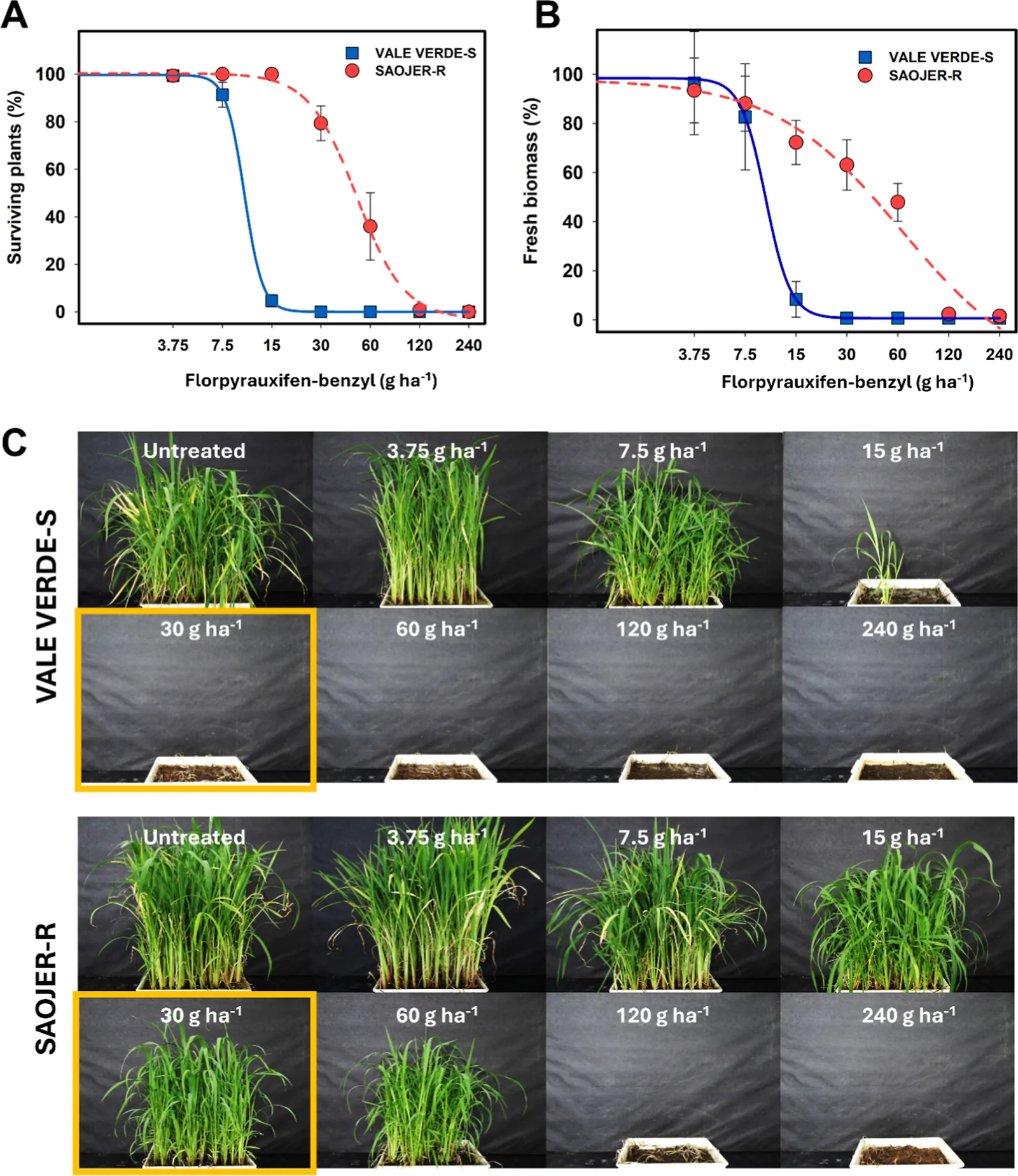
FPB survival dose–response curve. Number of survivors (graph in panel A and images in panel C) and fresh biomass (graph in panel B) of SAOJER-R and VALE VERDE-S *E. crus-galli* biotypes at different FPB doses. The yellow box highlights the label rate. Vertical bars indicate the confidence interval (95% interval).

### Effect of CYP450 and GST Inhibitors on FPB Resistance

Dose–response curves to evaluate the effect of metabolism inhibitors were carried out in the greenhouse (28 ± 5 °C). The experiment was carried out in a 2 × 8 × 3 factorial scheme with a completely randomized design. Factor A consisted of the biotypes SAOJER-R and VALE VERDE-S. Factor B consisted of the FPB doses of 0, 3.75, 7.5, 15, 30, 60, 120, and 240 g a.i. ha^–1^. Factor C consisted of the inhibitors malathion (CYP450) or NBD-Cl (GST) and an inhibitor-untreated check. Malathion was sprayed at 1500 g a.i. ha^–1^ 2 h before the herbicide, while the NBD-Cl (4-chloro-7-nitrobenzofurazan) was applied 48 h before at 270 g a.i. ha^–1^ diluted in a 1:1 acetone/water solution. The experimental unit was a 200 mL pot with one plant. Each treatment had five replicates. The application conditions and plant growth were the same as described before. Herbicide efficacy was evaluated at 14 and 35 DAT using percentage visual rating (scoring plants from 0 to 100, where 0 means no injury and 100 means dead), and fresh biomass was measured at 35 DAT. The data were analyzed as described previously. The experiment was carried out twice. Levene’s test of variance equality was used to compare the experiment runs.

### Effect of Temperatures on FPB Resistance

Dose–response curves with the two biotypes SAOJER-R and VALE VERDE-S were performed at temperatures of 35/30 °C (high temperature) or 25/20 °C (warm temperature) day/night in growth chambers. The humidity was ∼70%, and plants were under 14 h of light at 300–400 μmol m^–2^ s^–1^. The plants were kept at the treatment temperature for the entire experiment duration. The herbicide doses, plant growth, and spray conditions were the same as described in the previous section. Each treatment had five replicates, and the experiment was carried out twice. The evaluations consisted of herbicide efficacy using percentage visual rating and fresh biomass at 35 DAT. The data were analyzed as described previously.

### Metabolism Analysis with LC–MS/MS

The two *E. crus-galli* biotypes (resistant and susceptible) and cultivated rice (*O. sativa* cv. M206) were used to analyze the amount of FPB (parent compound—molecular weight 439.24) and FPA (active compound—molecular weight 349.12) by LC–MS/MS. The experiment was conducted in a growth chamber at 35/30 °C, and the plants were grown and sprayed as described previously at 30 g a.i. ha^–1^ of FPB commercial formulation. The plants were sampled at 8, 24, 72, 120, and 168 h after treatment (HAT). Additionally, at 72 and 120 HAT, samples that were sprayed with malathion (1500 g a.i. ha^–1^) 2 h before the herbicide were also collected. Each treatment had four replicates, and each replicate consisted of two plants. We acknowledge that this number of replicates may be limited.

The entire aboveground biomass (two plants per replicate) was collected, weighed, and washed three times in different pots with 1:1 ethanol/water. The plants were placed in 50 mL conical tubes and ground using liquid nitrogen and a pestle. Immediately, acetonitrile +0.1% formic acid was added at a 2:1 volume/mass ratio. A mechanical homogenizer was used to homogenize the samples for 50 s. The tubes were centrifuged at 3803*g* for 20 min at 15 °C. The supernatant was collected and filtered using hydrophobic PTFE syringe filters, pore size 0.22 μm and diameter 13 mm, into the vials. The LC–MS/MS system consisted of a Nexera X2 UPLC with 2 LC-30AD pumps, a SIL-30AC MP autosampler, a DGU-20A5 Prominence degasser, a CTO-30A column oven, and SPD-M30A diode array detector coupled to an 8040-quadrupole mass-spectrometer. For FPB, the MS was in the positive mode with a MRM optimized for 440.9 > 91.0 and set for 100 ms dwell time with a Q1 prebias of −30.0 V, a collision energy of −25.0 V, and a Q3 prebias of −16.0 V. For FPA, the MS was in the positive mode with a MRM optimized for 348.9 > 268.0 and set for 100 ms dwell time with a Q1 prebias of −24.0 V, a collision energy of −36.0 V, and a Q3 prebias of −27.0 V. The samples were chromatographed on a 100 × 4.6 mm Phenomenex Kinetex 2.6 μm F5 (pentafluoro) column maintained at 40 °C. Solvent A consisted of water with 0.1% formic acid, and solvent B was MeOH with 0.1% formic acid. The solvent gradient was: 0 min 30% solvent B; 4.5 min 55% solvent B; 5.6 min 95% solvent B; 9 min 95% solvent B; 9.2 min 30% solvent B; 11 min 30% solvent B. The flow rate was set at 0.4 mL min^–1^, and each sample was analyzed with a 5 μL injection volume. Calibration curves were made using 3, 10, 100, and 333 pg of FPB and FPA technical grade standards (Figure S1). The data were analyzed by ANOVA after checking assumptions. The comparison of means at each time point between susceptible, resistant, and rice was performed through the Tukey test (*p* ≤ 0.05). The means comparison between with and without malathion was through a *t*-test (α = 0.05).

### RNA Sequencing Analysis

The two *E. crus-galli* biotypes, SAOJER-R (resistant) and VALE VERDE-S (susceptible), were grown in a growth chamber under two temperature conditions, 25/20 °C and 35/30 °C day/night under 14 h of light. The plants were sprayed at 30 g a.i. ha^–1^ of FPB. The plant tissue collected was a segment of the two youngest leaves plus the meristem from four conditions: untreated at 35/30 °C, 24 HAT at 35/30 °C, 72 HAT at 35/30 °C, and 24 HAT at 25/20 °C. Each treatment had three replicates, and each replicate consisted of three plants. The RNA was extracted with RNeasy Plant Mini Kit (Qiagen) following the manufacturer’s protocol. During the extraction protocol, the samples were treated with DNase I (Invitrogen, Thermo Fisher Scientific, USA) to remove potential genomic DNA contamination. The RNA was sequenced at Novogene (California, USA) using Illumina Novaseq 6000 pair-ended 150 bp (6 GB raw data per sample).

The raw data were processed for adapter trimming and quality filtering using *fastp* v. 0.23.2 package.^33^ The processed reads were aligned to the last version of *E. crus-galli* genome available^13^ using the *HISAT2* v. 2.2.1 package.^34^ The alignment files from *HISAT2* and the genome annotation file were used to count the mapped reads using the *featureCounts* command from *Subread* v. 2.0.1 package.^35^ The analysis of read counts per contig and differentially expressed genes (DEGs) was done with the *DESeq2* package^36^ in *R* software. In *DESeq2* package, a minimal prefiltering was performed to keep only contigs that have at least 10 reads total. The Log2FoldChange (LFC) cutoff used was 1 and −1, which means twice upregulated and downregulated, respectively, and a false-discovery rate alpha ≤0.05 (adjusted p-value). The contrasts of interest built to analyze DEGs were “*R* vs *S* untreated 35/30 °C”, “*R* vs *S* sprayed 35/30 °C (24 HAT)”, “*R* vs *S* sprayed 25/20 °C (24 HAT)”, and “*R* vs *S* sprayed 35/30 °C (72 HAT)”.

The annotation of upregulated and downregulated contigs was performed using two strategies: the first was *HMMER* v.3.3.2 package^37^ and *P-famA* database (https://www.ebi.ac.uk/interpro/download/Pfam/), and the second was *BLASTp* v.2.12.0 ^38^ and UniProt database Swiss-Prot (https://www.uniprot.org/help/downloads) with maximum e-value of 1 × 10^–5^. The annotation was kept when the protein family annotated by the *Pfam*-*A* database was in accordance with the annotation from Swiss-Prot. Among the pool of DEGs for each contrast, we searched for the known gene families involved in herbicide metabolism *CYP*, *GST*, *UGT*, *ABC*, and *AKR*, in addition to *PER* (peroxidase) and auxin-responsive genes. Auxin-responsive indole-3-acetic acid (*IAA*), auxin efflux carrier (*PIN*), and ARF genes were searched among the differentially downregulated genes.

### Validation of RNA-seq with qPCR

A different set of plants from SAOJER-R and VALE VERDE-S biotypes was grown in a growth chamber at 35/30 °C for qPCR validation of the RNA-seq candidate genes. Samples of untreated and 24 HAT (30 g a.i. ha^–1^ of FPB) were collected. Plant growth and spray conditions were the same as described in previous sections. Each treatment had three biological replicates, and each replicate consisted of two plants. The RNA of the samples was extracted with Concert (Invitrogen) reagent protocol following the manufacturer’s specifications. The samples were treated with DNase I (Invitrogen) to eliminate DNA contaminants. cDNA synthesis was performed using the M_MLV reverse transcriptase (Invitrogen), according to the manufacturer’s protocol. The reactions were performed in a QuantStudio 3 Real-Time PCR System (ThermoFisher) using the PowerUp SYBR Green Master Mix (ThermoFisher) according to the manufacturer’s protocol. Each biological sample was performed with three technical replicates. The housekeeping genes tested were the translation initiation factor (*eIF4B1*), glyceraldehyde-3-phosphate dehydrogenase (*GAPDH*), rubisco (*RUB*), and 28S rRNA (28S). The most stable gene was *eIF4B1*, according to a comprehensive gene stability analysis in RefFinder (https://www.ciidirsinaloa.com.mx/RefFinder-master/?type=reference) and was utilized as a normalizer in relative expression (Figure S2). The target candidate genes were AH01.3668, AH08.1281, BH09.2162, CH01.634, CH01.638, BH04.1623, AH02.1632, BH01.4421, BH02.4558, BH02.4560, BH05.515, BH05.3318, CH01.480 + BH03.3871, AH03.690 + BH03.814, BH01.674, BH03.2759 + CH03.2963, BH03.3274, CH03.904, AH02.477, BH08.936, CH07.703, AH09.542, BH05.2320, BH09.1246, CH09.664, AH01.2685, and AH01.2477. Three primers targeted two genes simultaneously due to their high sequence similarity: CH01.480 + BH03.3871, AH03.690 + BH03.814, and BH03.2759 + CH03.2963. The primer sequences were designed utilizing software Primer3Plus (https://www.bioinformatics.nl/cgi-bin/primer3plus/primer3plus.cgi) and are listed in Table S1. The relative expression was calculated using 2^–(ΔΔCt)^ method. The data were submitted to ANOVA, and the averages were compared by the Tukey test 5%.

### Characterization of *CYP* Candidate Genes

A phylogenetic tree was performed utilizing the *CYP* candidate genes and 251 CYP450 enzyme protein sequences from plant CYP450 databases (https://p450.biodesign.ac.cn/; https://erda.dk/public/vgrid/PlantP450/index.html; https://www.arabidopsis.org/browse/genefamily/p450.jsp; https://drnelson.uthsc.edu/wp-content/uploads/sites/130/resources/biblioD.html.^39^ The sequences were aligned by utilizing the MUSCLE algorithm with default parameters. The phylogenetic trees were built utilizing the protein alignment and neighbor-joining method. The bootstrap consensus tree was inferred from 5000 replicates. The evolutionary distances were computed by using the Poisson correction method and are in units of the number of amino acid substitutions per site. The *E. crus-galli* genome^13^ was utilized to make a karyotype with the *Rideogram* package^40^ in *R*, showing the gene density and the location of *CYP* candidate genes.

## Results

### Screening of *E. crus-galli* Biotypes with FPB and Resistance Confirmation

Of 20 biotypes screened, nine had at least one plant surviving the application of the FPB at 25 g a.i. ha^–1^ (Table 1). The resistance profile background of the surviving biotypes is diverse, including two biotypes that are susceptible to all herbicides already tested. All other surviving biotypes are resistant to ALS inhibitors; one is additionally resistant to quinclorac and cyhalofop-butyl, and one also showed resistance to quinclorac and fenoxaprop-p-ethyl, according to previous studies.^3,4,6,8,41^ The biotypes with the highest FPB survival rates were ANTAGO-02 (42.8%), 354 (39.6%), MOSTS-51 (19.6%), and SAOJER-01 (19.6%) (Table 1). At the G1 seed generation, the dose sprayed was the maximum FPB label rate (30 g a.i. ha^–1^), and all biotypes selected had at least two surviving plants. The biotypes ANTAGO-02 (40%), 354 (45.45%), MOSTS-51 (48.83%), and SAOJER-01 (96.15%) showed the highest survival rates again. The biotype SAOJER-01 (called SAOJER-R from here) had 53% of plants surviving to 60 g a.i. ha^–1^ of FPB at G2. The dose–response curves showed that the biotype SAOJER-R is resistant to FPB, with an ED_50_ higher than 44 g a.i. ha^–1^, while the susceptible VALE VERDE-S had ED_50_ around 10 g a.i. ha^–1^, resulting in a resistance factor (RF) of 4.7 for the number of surviving plants and 4.5 for the fresh biomass (Table 2; Figure 1A–C).

### Effect of CYP450 and GST Inhibitors on FPB Resistance

The dose–response curves of FPB without a metabolic inhibitor showed similar visual injury in both susceptible and resistant biotypes at 14 DAT, both developing auxin-like symptoms (epinasty and twisting) and an RF of 1.6 for SAOJER-R (Table 3). However, from 14 DAT onward, a regrowth ability was observed in SAOJER-R, while VALE VERDE-S died. At 35 DAT, the dose–response curves showed a difference between the biotypes, with an RF of 3.8 for SAOJER-R (Table 3; Figure 2A,C). The evaluation at 35 DAT, instead of 21 or 28 DAT, was important to capture the regrowth ability of the resistant biotype in the greenhouse.

**Table 3:** Metabolic Inhibitors and FPB Dose–Response Curves^a^ ^,^ ^b^

					*e*(ED_50_)		
biotypes	inhibitor	*b*	*d*	*e*(ED_50_)	lower limit	upper limit	RF
		herbicide efficacy (14 DAT)					
VALE VERDE-S	no	–10.45^ns^	100.05*	8.38*	4.49	12.26	
SAOJER-R	no	–4.19*	86.75*	13.35*	11.99	14.72	1.59 ns
VALE VERDE-S	malathion	–5.50*	100.02*	9.35*	8.38	10.32	
SAOJER-R	malathion	–4.47*	91.91*	10.56*	9.44	11.67	1.13 ns
VALE VERDE-S	NBD-Cl	–4.67*	98.97*	10.06*	9.06	11.06	
SAOJER-R	NBD-Cl	–3.78*	92.62*	19.07*	16.90	21.24	1.89*
		herbicide efficacy (35 DAT)					
VALE VERDE-S	no	–6.65*	100.00*	9.63*	8.26	11.00	
SAOJER-R	no	–2.14*	100.00*	55.42*	45.01	65.84	5.76*
VALE VERDE-S	malathion	–6.08*	100.00*	9.24*	8.02	10.45	
SAOJER-R	malathion	–2.42*	100.00*	22.20*	18.80	25.61	2.40*
VALE VERDE-S	NBD-Cl	–4.89*	100.00*	12.09*	10.77	13.42	
SAOJER-R	NBD-Cl	–3.20*	100.00*	53.40*	46.38	60.43	4.41*
		fresh biomass (g)					
VALE VERDE-S	no	18.46^ns^	66.35*	7.72*	4.47	10.96	
SAOJER-R	no	1.42*	50.50*	29.32*	21.68	36.96	3.80*
VALE VERDE-S	malathion	6.54^ns^	56.06*	8.05*	7.26	8.85	
SAOJER-R	malathion	1.92*	55.27*	12.00*	9.73	14.28	1.49*
VALE VERDE-S	NBD-Cl	6.14*	40.96*	11.11*	9.03	13.19	
SAOJER-R	NBD-Cl	1.79*	50.21*	27.94*	22.05	33.84	2.51*

a Three-parameter log-logistic model for the FPB dose–response curve with two *E. crus-galli* biotypes, evaluating the effect of metabolic inhibitors malathion (CYP450) and NBD-Cl (GST) (greenhouse at 28 ± 5 °C).

b *b*: Curve slope at the inflection point; *d*: upper limit; *e*(ED_50)_: Effective dose to reduce 50% of the variable. RF: resistance factor based on ED_50._ Asterisk (*) or ^ns^ indicates the parameter significance fitting the log–logistic model (*p* ≤ 0.05).

**Figure 2:**
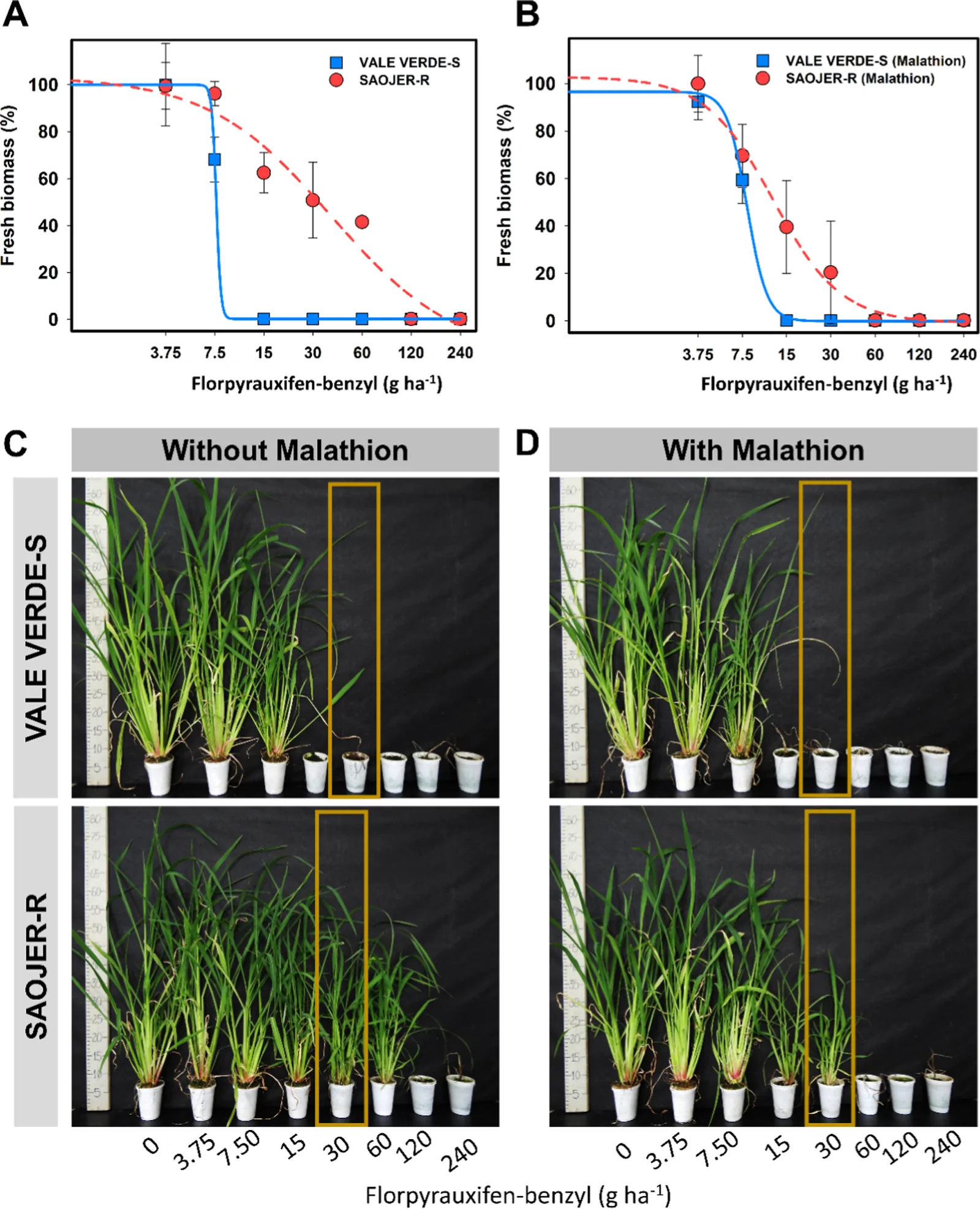
Effect of CYP450 metabolic inhibitor on the FPB dose–response curve for SAOJER-R and VALE VERDE-S *E. crus-galli* biotypes. Dose–response curve with FPB alone (graph in panel A and images in panel C), and FPB with previous application of malathion (graph in panel B and images in panel D). The yellow box highlights the label rate. Vertical bars indicate the confidence interval (95% interval).

Application of the CYP450 inhibitor malathion reduced the RF of the SAOJER-R. The biomass ED_50_ of SAOJER-R was reduced from 29.3 without CYP450 inhibitor to 12 g a.i. ha^–1^ of FPB with the inhibitor, and consequently the RF was reduced from 3.8 to 1.5 (Table 3; Figure 2B,D). The application of the GST inhibitor NBD-Cl did not reverse the resistance. Although there was a reduction in RF from 3.8 to 2.5 when comparing the biotypes, the ED_50_ of the SAOJER-R biotype with and without the GST inhibitor did not differ. The RF reduction was because the susceptible biotype showed a higher ED_50_ when sprayed with the GST inhibitor (Table 3). Overall, the application of metabolic inhibitors did not cause phytotoxicity to plants, except for NBD-Cl in the susceptible biotype (Tables S2 and S3).

### Effect of Temperature on FPB Resistance

The FPB injuries at 7, 14, and 35 DAT on SAOJER-R were higher at 25/20 °C than at 35/30 °C (Table 4; Figure 3A–D). For the herbicide efficacy at 35 DAT, the RF of SAOJER-R increased from 1.5 to 7.5 at 25/20 °C and 35/30 °C, respectively, and the RF based on fresh biomass was 2.4 and 5.2 at temperatures of 25/20 °C and 35/30 °C, respectively. The ability of the resistant biotype SAOJER-R to regrow at the label rate of 30 g a.i. ha^–1^ occurred at both temperature conditions, but it was faster at 35/30 °C. Increasing the temperature also allowed the susceptible biotype VALE VERDE-S to increase its survival to FPB, surviving up to 7.5 g a.i. ha^–1^ at 25/20 °C and 15 g a.i. ha^–1^ at 35/30 °C, while the resistant biotype SAOJER-R survived up to 30 and 120 g a.i. ha^–1^, respectively (Figure 3A–D).

**Table 4:** FPB Dose–Response Curves at Two Different Temperatures^a^ ^,^ ^b^

					*e*(ED_50_)		
diotypes	day/night temp	*b*	*d*	*e*(ED_50_)	lower limit	upper limit	RF
		herbicide efficacy (7 DAT)					
VALE VERDE-S	25/20 °C	–13.07^ns^	70.00*	13.08*	3.30	22.86	
SAOJER-R	25/20 °C	–22.74^ns^	70.00*	15.52*	7.42	23.63	1.19 ns
VALE VERDE-S	35/30 °C	–2.93*	83.19*	11.36*	10.66	12.07	
SAOJER-R	35/30 °C	–3.73*	72.26*	21.57*	20.20	22.94	1.90*
		herbicide efficacy (14 DAT)					
VALE VERDE-S	25/20 °C	–4.73*	94.93*	17.19*	15.87	18.50	
SAOJER-R	25/20 °C	–5.81*	85.07*	21.23*	19.23	23.22	1.23*
VALE VERDE-S	35/30 °C	–4.11*	100.22*	9.99*	9.24	10.73	
SAOJER-R	35/30 °C	–2.27*	82.90*	28.35*	24.33	32.37	2.84*
		herbicide efficacy (35 DAT)					
VALE VERDE-S	25/20 °C	–5.26*	100.07*	19.32*	17.18	21.456	
SAOJER-R	25/20 °C	–2.73*	100.89*	28.97*	25.19	32.753	1.50*
VALE VERDE-S	35/30 °C	–3.60*	101.55*	14.53*	13.11	15.946	
SAOJER-R	35/30 °C	–2.10*	100.00*	109.74*	89.54	129.949	7.55*
		fresh biomass (g)					
VALE VERDE-S	25/20 °C	4.91*	37.40*	8.13*	7.21	9.05	
SAOJER-R	25/20 °C	2.62*	41.47*	19.32*	16.22	22.41	2.37*
VALE VERDE-S	35/30 °C	2.74*	32.84*	9.58*	7.57	11.59	
SAOJER-R	35/30 °C	0.92*	33.70*	49.99*	26.28	73.70	5.22*

a Three-parameter log-logistic model for the FPB dose–response curve with two *E. crus-galli* biotypes conducted in a growth chamber at 25/20 °C and 35/30 °C.

b *b*: curve slope at the inflection point; *d*: upper limit; *e*(ED_50)_: effective dose to reduce 50% of the variable. RF: resistance factor based on ED_50._ Asterisk (*) or ^ns^ indicates the parameter significance fitting the log–logistic model (*p* ≤ 0.05).

**Figure 3:**
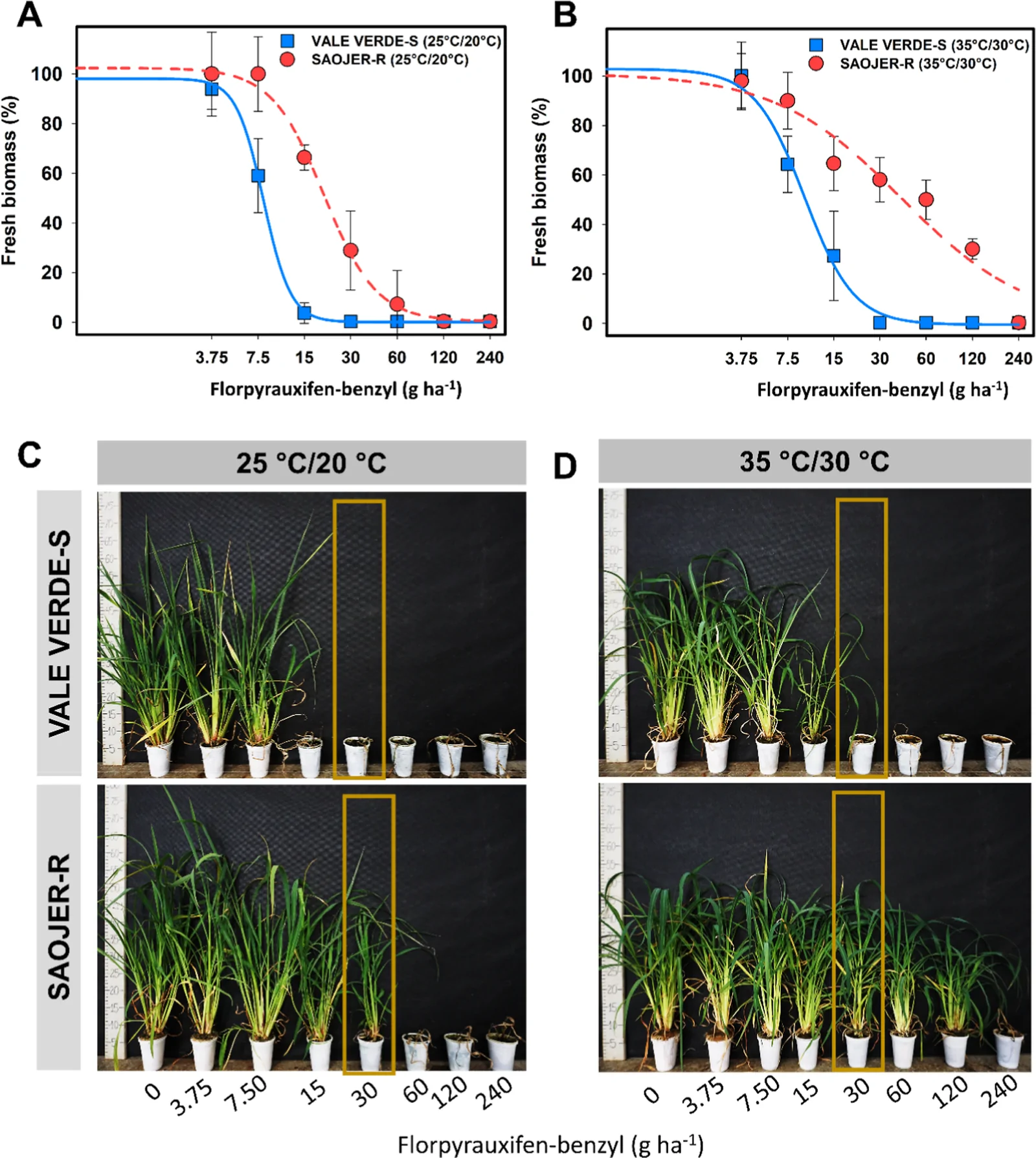
FPB dose–response curves at two different temperatures. Dose–response curve of FPB at 25/20 °C (graph in panel A and images in panel C), and 35/30 °C (graph in panel B and images in panel D) for SAOJER-R and VALE VERDE-S *E. crus-galli* biotypes. The yellow box highlights the label rate. Vertical bars indicate the confidence interval (95% interval).

### Metabolism Analysis with LC–MS/MS

The susceptible biotype VALE VERDE-S accumulated more FPB and FPA than the resistant biotype SAOJER-R and rice at all five time points evaluated at 35/30 °C. The VALE VERDE-S had 404 ppm of FPB at 8 HAT, while the SAOJER-R and rice had 93 and 37 ppm, respectively (Figure 4A). At 168 HAT, the VALE VERDE-S still had 87 ppm, while SAOJER-R and rice had 20 and 17 ppm, respectively. At 8 HAT, the amount of FPA, the active herbicide compound, detected in the VALE VERDE-S was 3216 ppm, compared to 888 ppm in SAOJER-R and 447 ppm in rice (Figure 4B). VALE VERDE-S, SAOJER-R, and rice decreased the FPA concentration across the time points; however, the susceptible biotype still showed 6.6 and 10 times more FPA than the resistant biotype at 24 and 72 HAT, for example. At 120 HAT, the biotype SAOJER-R had already metabolized all FPA, while VALE VERDE-S still had 223 ppm, and rice had 98 ppm in the plant. The amounts of FPB and FPA detected in SAOJER-R and rice were similar, indicating a similarity in detoxification speed or reduced uptake in rice (not evaluated in this study).

**Figure 4:**
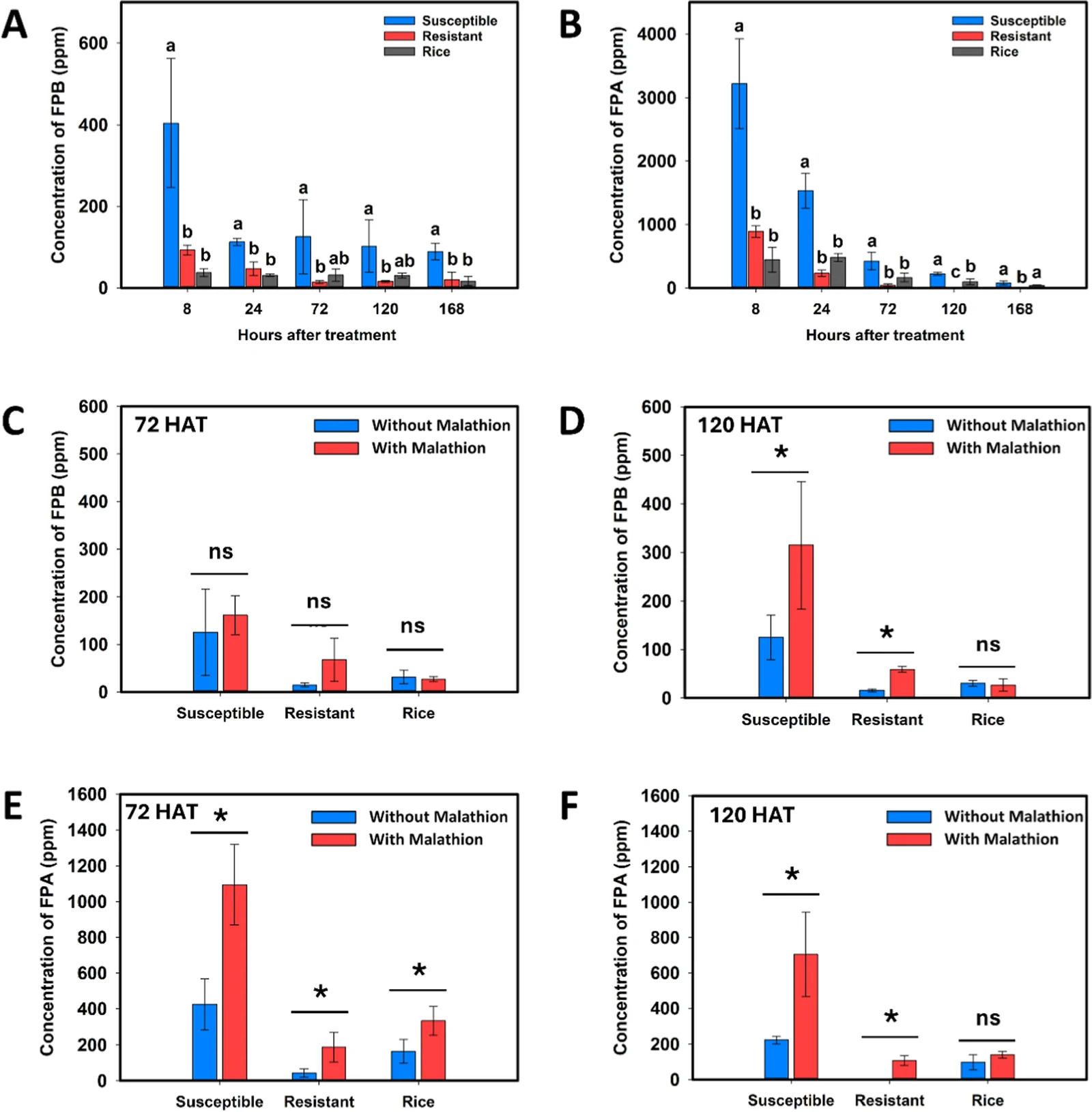
Quantification of FPB and FPA utilizing LC–MS/MS. Quantification of FPB (A) and FPA (B) in SAOJER-R and VALE VERDE-S *E. crus-galli* biotypes and rice at five time points. Quantification of FPB at 72 HAT (C) and 120 HAT (D), and quantification of FPA at 72 HAT (E) and 120 HAT (F) in the absence and presence of CYP450 inhibitor malathion, respectively. Letters compare *E. crus-galli* biotypes and rice averages within the same sampling time point, according to the Tukey test 5%. Asterisk (*) or ^ns^ compares treatments with and without malathion within the same biotype, according to the *t*-test (*p* ≤ 0.05). Vertical bars indicate the confidence interval (95% interval).

The presence of malathion, a CYP450 inhibitor, did not affect the amount of FPB at 72 HAT in any of the biotypes and rice (Figure 4C). However, the FPB amount increased 2.5 times in the susceptible and 3.8 times in the resistant biotype at 120 HAT when malathion was sprayed before the herbicide, while rice was not affected (Figure 4D). The amount of FPA at 72 HAT was 2.6, 4.5, and 2.0 times higher in VALE VERDE-S, SAOJER-R, and rice, respectively, when sprayed with malathion in comparison to malathion-untreated plants (Figure 4E). At 120 HAT, malathion still increased the amount of FPA detected in VALE VERDE-S and SAOJER-R but not in rice (Figure 4F). In the absence of malathion, the SAOJER-R biotype metabolized all of the FPA at 120 HAT, but when malathion was sprayed, it still had 108 ppm in the plant. The effect of malathion in increasing the amount of FPA in the susceptible biotype is expected, considering that it also has CYP450 enzymes acting on herbicide detoxification; however, the activity in the susceptible biotype is less efficient than in the resistant.

### RNA Sequencing Analysis

The alignment of reads to the genome and differential expression analysis resulted in 72,262 genes with nonzero counts. There were ∼650 upregulated and ∼550 downregulated genes in each of the contrasts “*R* vs *S* untreated 35/30 °C (24 HAT)”, “*R* vs *S* sprayed 25/20 °C (24 HAT)”, and “*R* vs *S* sprayed 35/30 °C (24 HAT)”. The contrast “*R* vs *S* sprayed 35/30 °C (72 HAT)” showed almost twice as many upregulated and downregulated genes as the contrasts at 24 HAT. The Venn diagram indicates that 404 upregulated genes in SAOJER-R were shared by all contrasts, 36 genes were shared by contrasts when FPB was sprayed, and 49 genes were shared by contrasts at high temperature (Figure 5). There were 329 genes downregulated in SAOJER-R shared by all four contrasts, 25 genes shared by contrasts where FPB was sprayed, and 53 genes shared by contrasts at high temperature (Figure 5).

**Figure 5:**
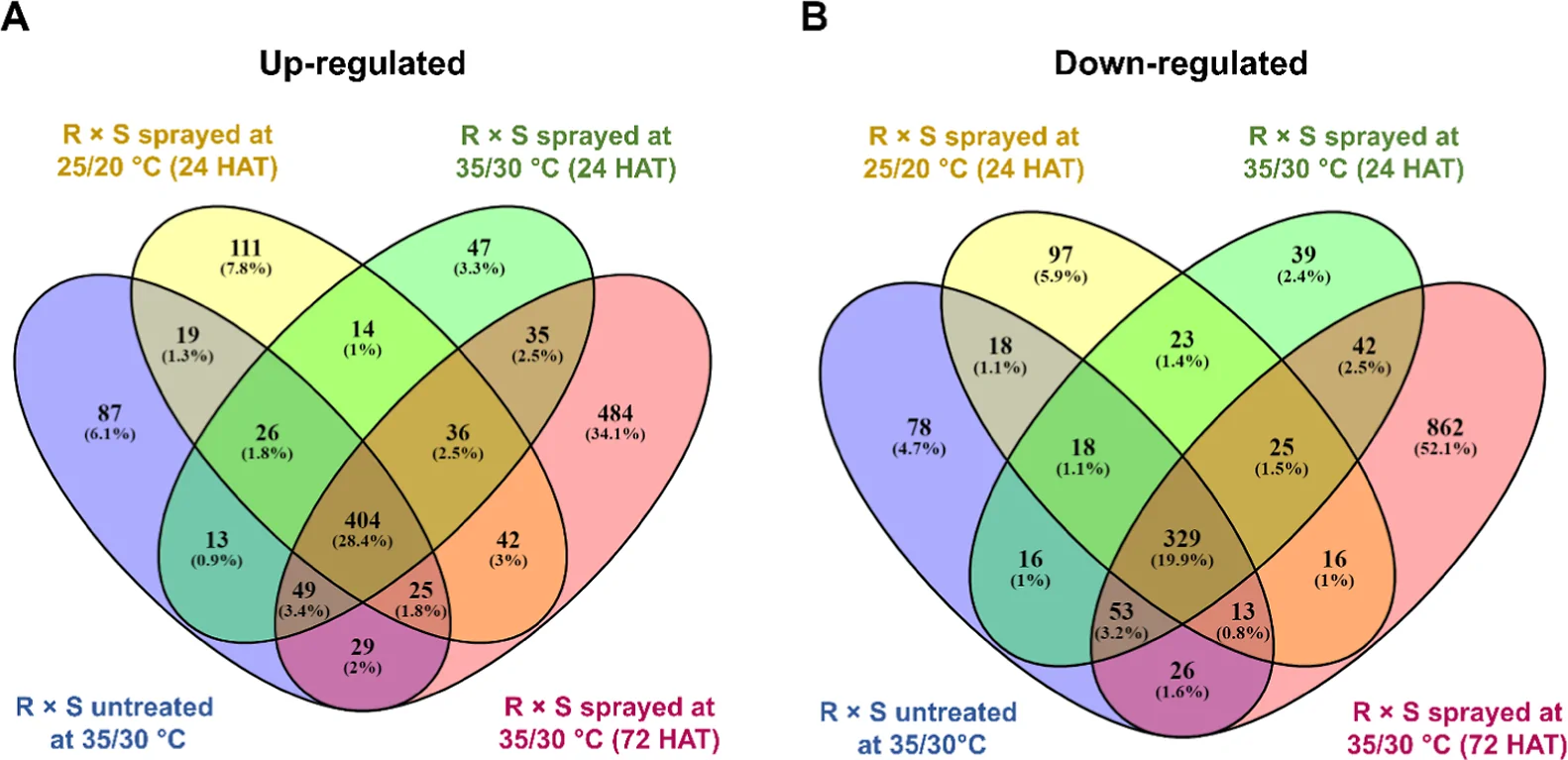
Venn diagram of genes upregulated (A) and downregulated (B) shared by all four contrasts studied.

The analysis of all contrasts showed 27 genes from the *CYP* family upregulated in SAOJER-R (Figure 6). The genes AH08.1281, BH09.2162, and CH01.638 were the only ones upregulated in both contrasts “*R* vs *S* sprayed 25/20 °C (24 HAT)” and “*R* vs *S* sprayed 35/30 °C (24 HAT)”, but also in “*R* vs *S* untreated 35/30 °C”. The upregulation of genes at both temperatures, 25/20 °C and 35/30 °C, is important because the SAOJER-R biotype is resistant in both conditions, even though with different RF. The Log2FoldChange of AH08.1281 ranged from 5.85 to 7.56, BH09.2162 from 2.91 to 4.57, and CH01.638 from 2.08 to 2.58 (Figure 6). The genes AH08.1281 and BH09.2162 showed increased expression at 35/30 °C when compared to 25/20 °C. The differential expression of these three *CYP* genes was not induced by the FPB, considering they were also upregulated in the untreated contrast “*R* vs *S* untreated 35/30 °C”. AH08.1281 and BH09.2162 were also upregulated in the contrast “*R* vs *S* sprayed 35/30 °C (72 HAT)”, but not CH01.638 (Figure 6).

**Figure 6:**
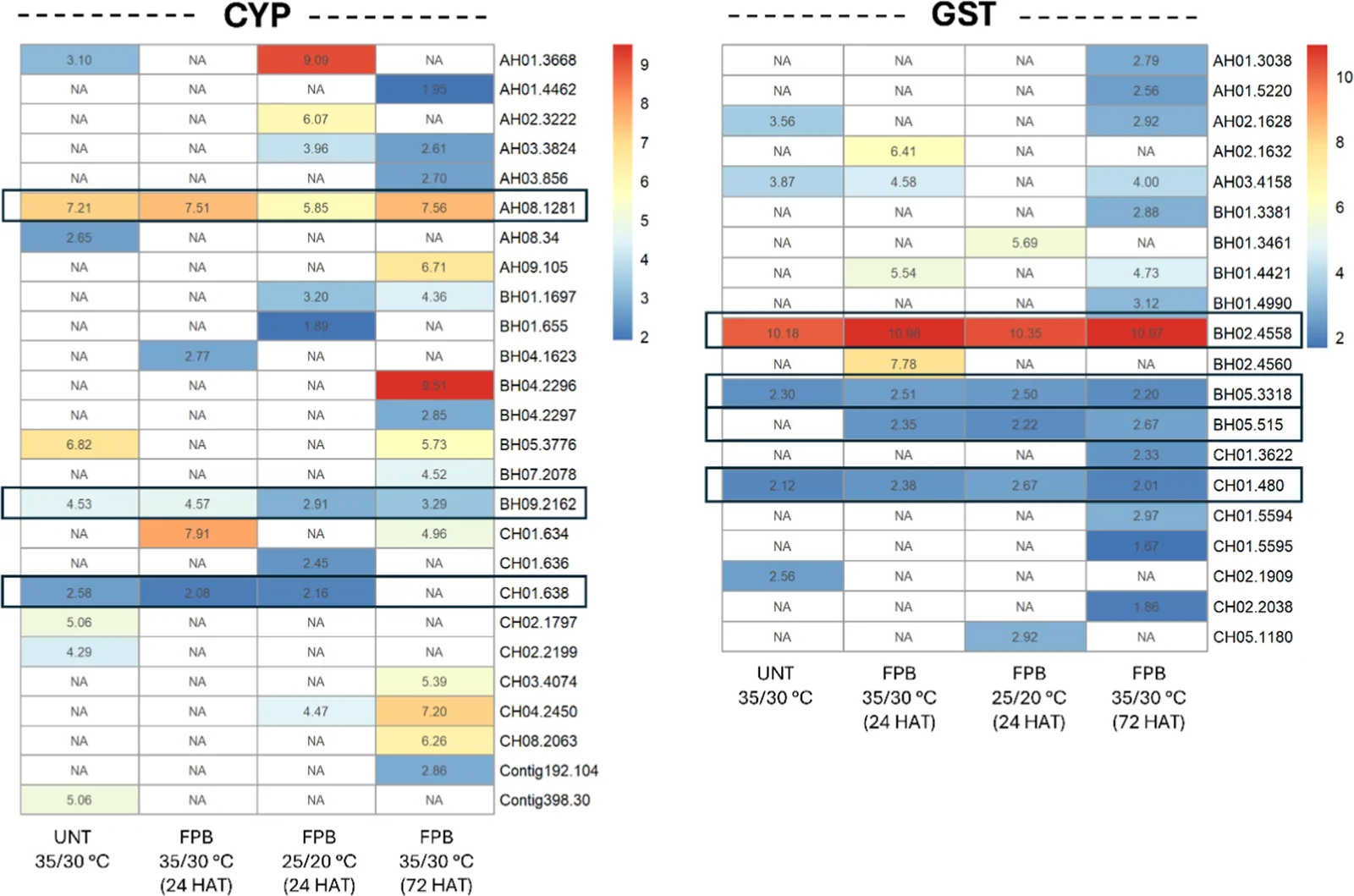
*CYP* and *GST* genes upregulated in the resistant biotype SAOJER-R compared to the susceptible VALE VERDE-S under four conditions (untreated (UNT) and treated with FPB). Boxes highlight the genes upregulated in both contrasts, “*R* vs *S* sprayed 35/30 °C (24 HAT)” and “*R* vs *S* sprayed 25/20 °C (24 HAT)”. “NA” indicates no differential expression in that condition.

Considering all four contrasts, 21 upregulated genes from the GST family were identified (Figure 6). The genes BH02.4558, BH05.3318, BH05.515, and CH01.480 were upregulated in both contrasts “*R* vs *S* sprayed 25/20 °C (24 HAT)” and “*R* vs *S* sprayed 35/30 °C (24 HAT)”, with Log2FoldChange ranging from 10.2 to 11.0, 2.2 to 2.5, 2.2 to 2.7, and 2.0 to 2.7, respectively. The *UGT* gene family showed 11 genes upregulated, but only BH01.674, BH03.2759, BH03.3274, and CH03.2963 were upregulated in both contrasts of interest, with Log2FoldChange ranging from 2.1 to 5.4, 4.2 to 7.9, 3.2 to 7.7, and 3.0 to 5.0, respectively (Figure 7). The contrast “*R* vs *S* sprayed 35/30 °C (72 HAT)” showed more *CYP*, *GST*, and *UGT* genes upregulated when compared to the contrast from 24 HAT. Other gene families involved in metabolism also had genes upregulated in the SAOJER-R when compared to VALE VERDE-S. The *ABC* and *AKR* families had five genes upregulated each, but three in both contrasts “*R* vs *S* sprayed 25/20 °C (24 HAT)” and “*R* vs *S* sprayed 35/30 °C (24 HAT)” (Figure 7). The *ABC* gene CH07.703 showed a Log2FoldChange of 9.0 in all four contrasts. The *PER* family had seven genes upregulated, but only three in both contrasts (Figure 7).

**Figure 7:**
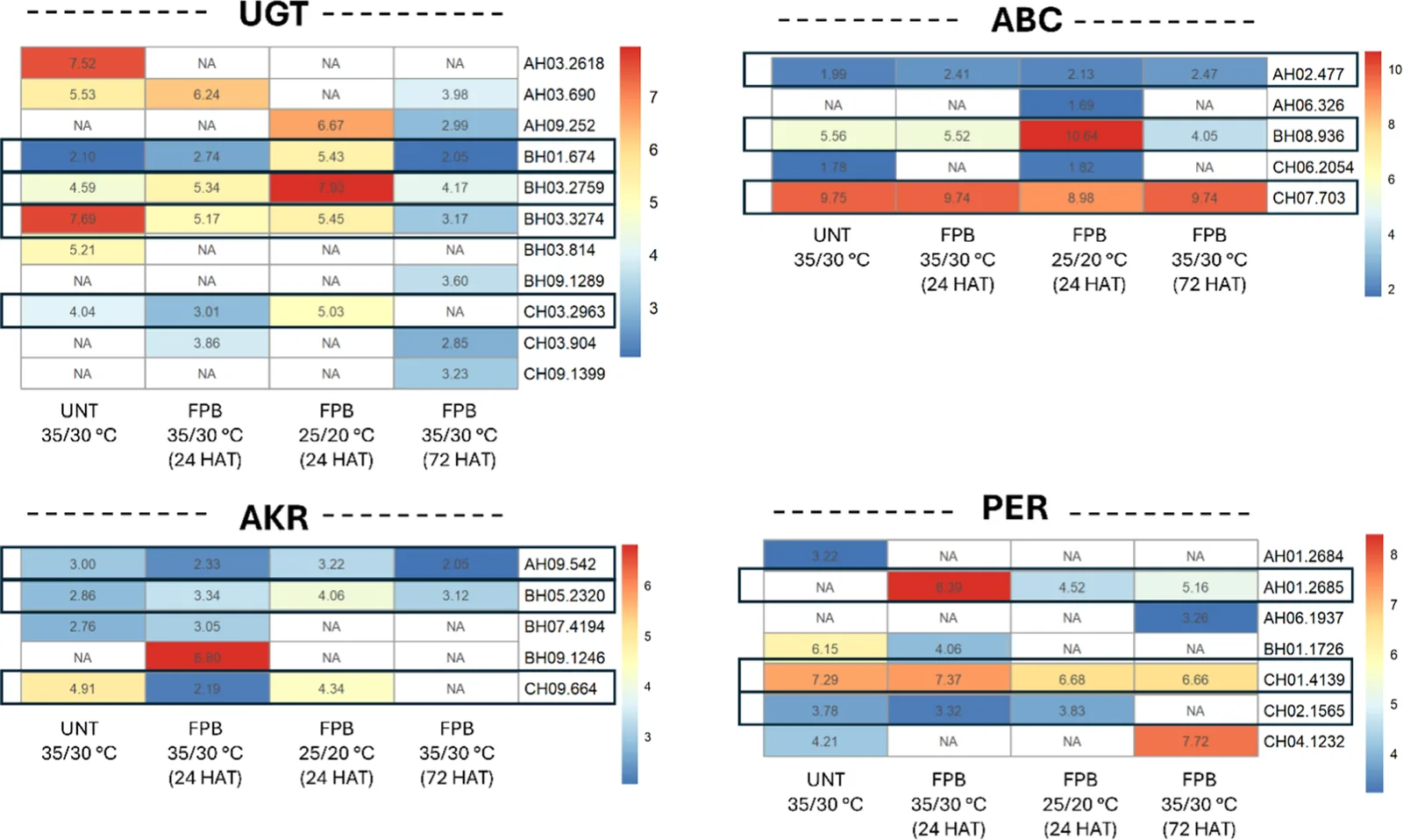
*UGT*, *ABC*, *AKR*, and *PER* genes upregulated in the resistant biotype SAOJER-R compared to the susceptible VALE VERDE-S under four conditions (untreated (UNT) and treated with FPB). Boxes highlight the genes upregulated in both contrasts, “*R* vs *S* sprayed 35/30 °C (24 HAT)” and “*R* vs *S* sprayed 25/20 °C (24 HAT)”. “NA” indicates no differential expression in that condition.

The differential expression of genes related to auxin response was also investigated. The genes AH01.2477, AH02.1335, AH07.925, AH08.1747, BH07.2465, CH03.3327, and BH01.2887 were the auxin-responsive genes upregulated and were annotated as *GH3*.*2*, *IAA4*, *IAA7*, *SAUR76*, *SAUR32*, *SAUR39*, and *LAX3*, respectively. However, none of those genes were upregulated in both contrasts “*R* vs *S* sprayed 25/20 °C (24 HAT)” and “*R* vs *S* sprayed 35/30 °C (24 HAT)” (Figure S3). The annotation of all selected upregulated genes is shown in Table S4. The BH08.2191 and CH05.1866 genes, annotated as *ARF* 21*-like* (*ARF21*) and *auxin efflux carrier component* 3*b* (*PIN3B*), respectively, were consistently downregulated in the resistant biotype compared to the susceptible in all four contrasts analyzed (Figure S4; Table S5). There was no *IAA* gene consistently downregulated in all four contrasts analyzed (Figure S4).

### Validation of RNA-seq Utilizing qPCR

The genes that were upregulated in both RNA-seq conditions, “*R* vs *S* sprayed 25/20 °C (24 HAT)” and “*R* vs *S* sprayed 35/30 °C (24 HAT) (Figures 8 and 9), and some upregulated in only one of the conditions (Figure S5) were selected for validation. Out of 17 main candidates, the expression of three *CYP*, two *GST*, four *UGT*, three *ABC*, two *AKR*, and one *PER* was higher in resistant SAOJER-R when compared to the susceptible VALE VERDE-S in qPCR (Figures 8 and 9). The candidate gene AH08.1281 from the *CYP* family showed 52 times higher expression in SAOJER-R untreated than VALE VERDE-S untreated; its expression increased in both biotypes after FPB treatment, but SAOJER-R still had 25 times higher expression than the susceptible treated (Figure 8). The *ABC* genes BH08.936 and CH07.703 also showed high differential expression in SAOJER-R compared with VALE VERDE-S, with expression levels up to 300-fold higher (Figure 9). Other genes with significant upregulation in SAOJER-R were the *GST* gene BH02.4558, the *UGT* genes BH03.3274 and BH03.2759 + CH03.2963, *AKR* gene CH09.664, and *PER* gene AH01.2685, with expression ranging (Figures 8 and 9).

**Figure 8:**
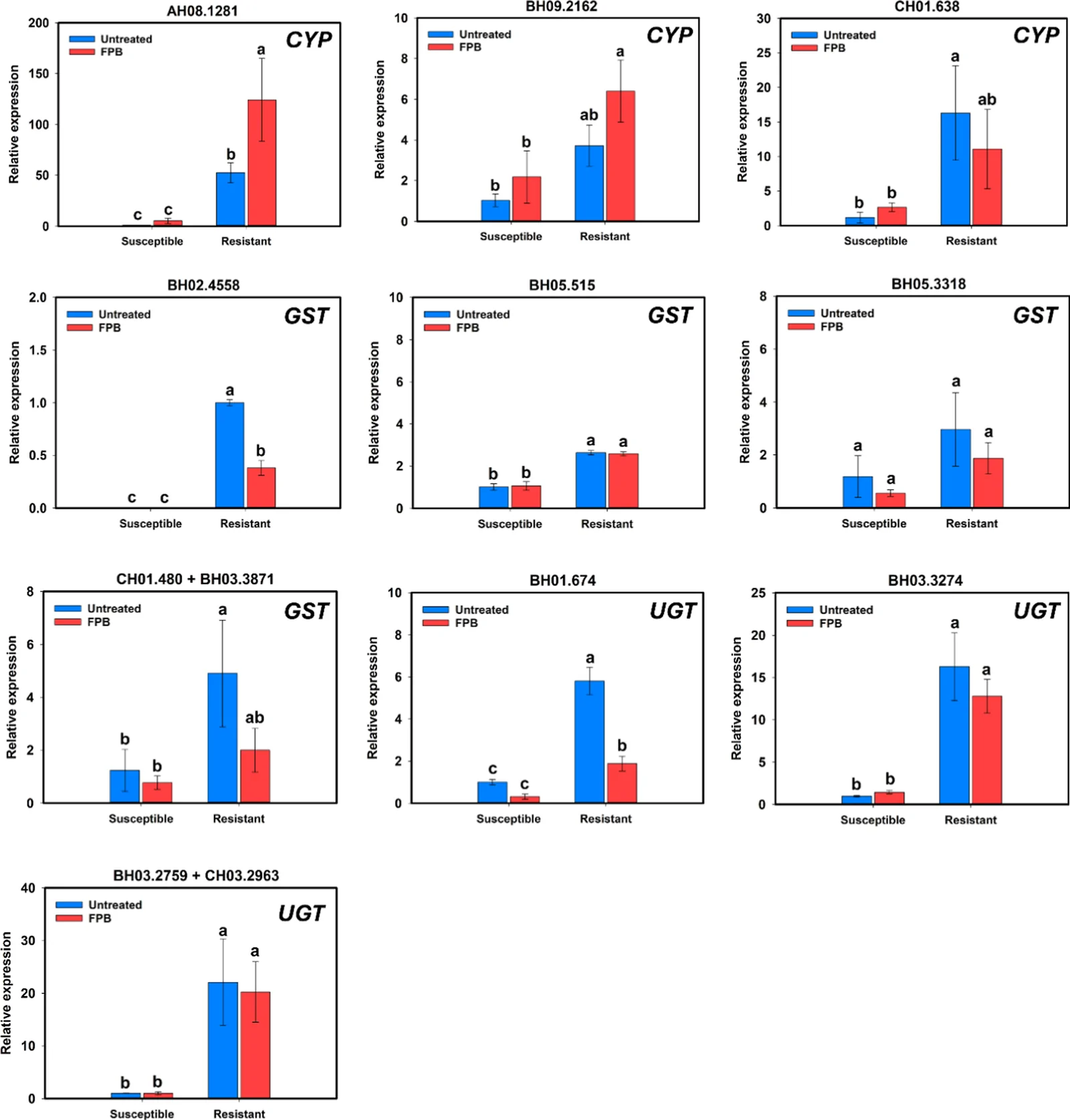
qPCR validation of *CYP*, *GST*, and *UGT* genes upregulated in the RNA-seq analysis. Relative expression of selected genes from *CYP*, *GST*, and *UGT* families upregulated under both temperature conditions 25/20 °C and 35/30 °C in the RNA-seq, utilizing SAOJER-R resistant and VALE VERDE-S susceptible *E. crus-galli* biotypes, untreated and treated with FPB, and collected at 24 HAT at 35/30 °C. Vertical bars indicate the confidence interval (95% interval).

**Figure 9:**
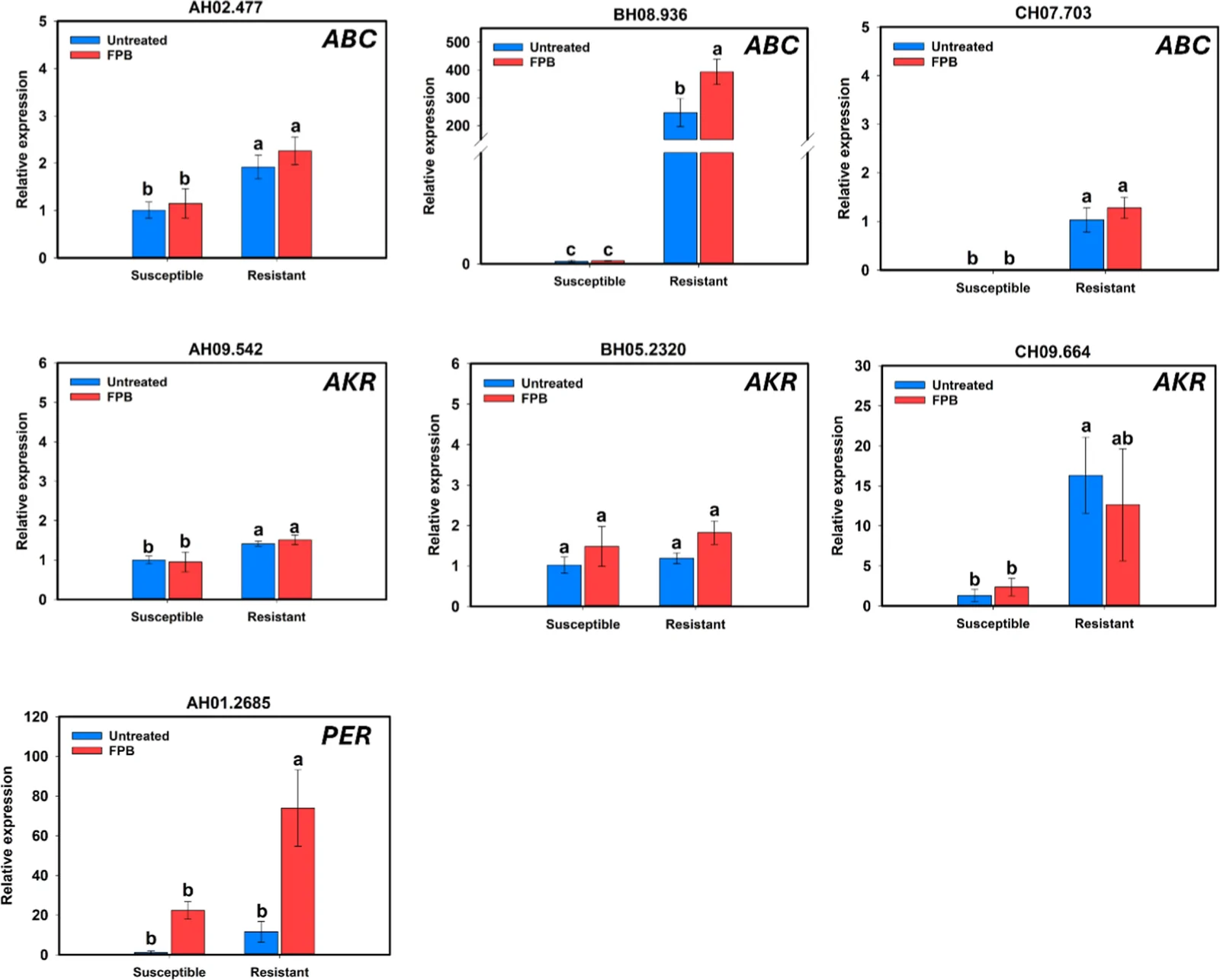
qPCR validation of *ABC*, *AKR*, and *PER* genes upregulated in the RNA-seq analysis. Relative expression of selected genes from *ABC*, *AKR*, and *PER* families upregulated under both temperature conditions 25/20 °C and 35/30 °C in the RNA-seq, utilizing SAOJER-R resistant and VALE VERDE-S susceptible *E. crus-galli* biotypes, untreated and treated with FPB, and collected at 24 HAT at 35/30 °C. Vertical bars indicate the confidence interval (95% interval).

### Characterization of *CYP* Candidate genes

The phylogenetic tree of the three *CYP* candidate genes compared to 251 other *CYP* sequences indicates that gene AH08.1281 is a *CYP71Z*-like (Figure 10A), BH09.2162 is a *CYP704C*-like (Figure 10B), and CH01.638 is a *CYP81A*-like, with more similarity to *CYP81A4* (Figure 10C). The complete phylogenetic tree is shown in Figure S6, while Figure 10 zooms in on where the candidate genes were located. The *E. crus-galli* karyotype indicates that the genes AH08.1281, BH09.2162, and CH01.638 are located in Chr 08 of subgenome A, Chr 09 of subgenome B, and Chr 01 of subgenome C, respectively (Figure S7).

**Figure 10:**
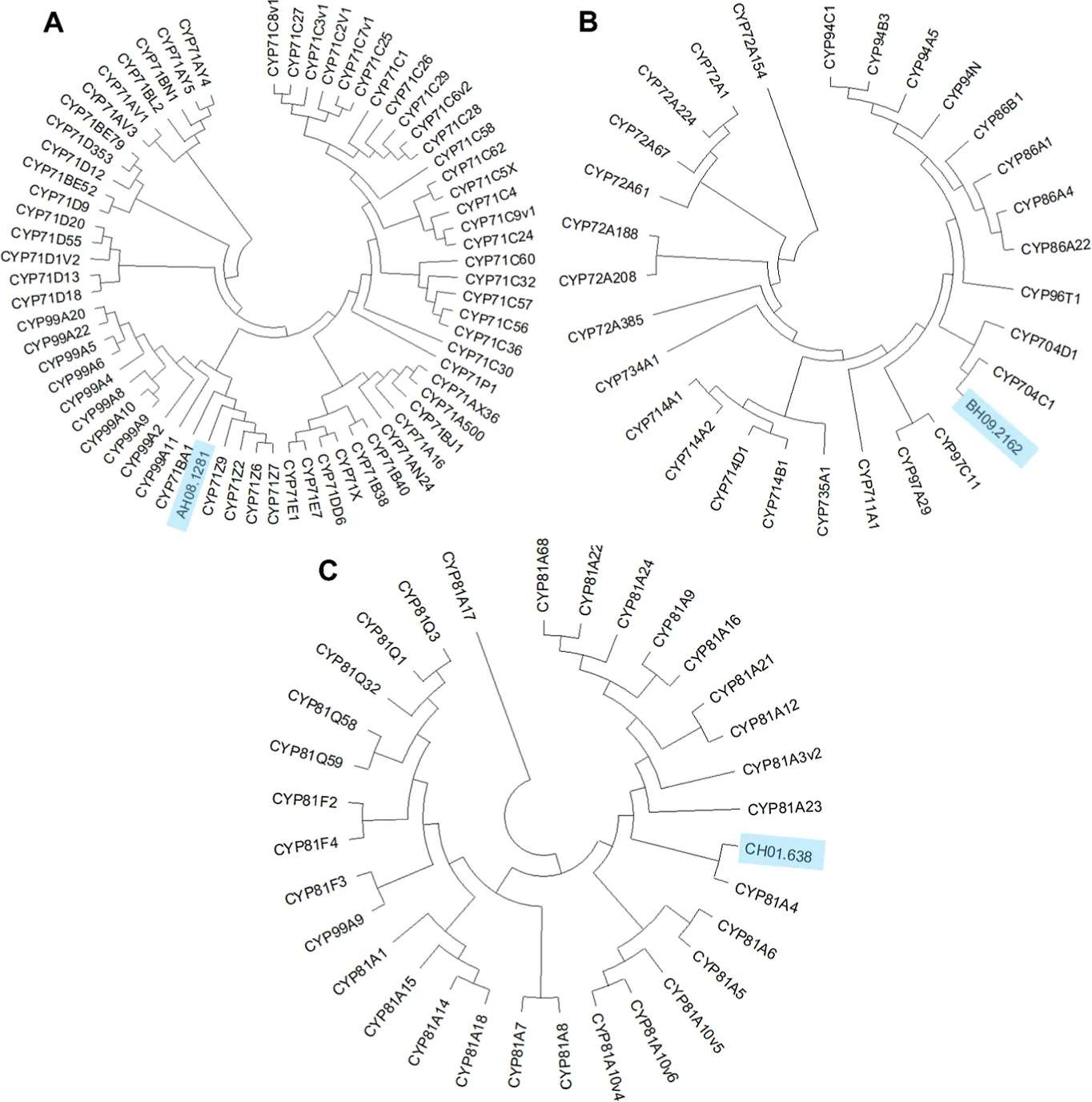
*CYP* gene family phylogenetic tree. Zoom in on the phylogenetic tree of *CYP* genes to annotate the candidate genes AH08.1281 (A), BH09.2162 (B), and CH01.638 (C), highlighted in blue.

## Discussion

None of the biotypes tested in this study had any prior history of the FPB application. Nevertheless, nine out of 20 biotypes had at least one plant surviving the herbicide label rate, and among them, four had at least a 20% survival rate. A possible explanation could be that previous selections imposed by other herbicides, such as penoxsulam, may have been selected for FPB resistance.^28^ The biotype SAOJER-R identified here as resistant to FPB has previously been selected for penoxsulam resistance due to *ALS* target site mutation Trp574Leu,^6^ and metabolic resistance to cyhalofop-butyl.^3^ In contrast, the biotype ANTAGO-02 is susceptible to all herbicides and had 43% of plants surviving FPB application, reinforcing that prior selection with other herbicides is not the only possible explanation and supporting the hypothesis of natural genetic variation. The efficacy of FPB on *Echinochloa* spp. control across the world has been variable. A study carried out in Korea found nonsatisfactory herbicide efficacy in 10% of 70 *E. crus-galli* biotypes and 32% survival among 71 *E. phyllopogon* biotypes.^42^ A screening of 170 *E. crus-galli* biotypes collected in midsouthern USA had four consistent surviving biotypes (2.3%),^27^ while a screening in Arkansas (USA) had more than 90% of herbicide efficacy at the label rate for all 152 biotypes evaluated.^22^

The SAOJER-R RF of 4 to 5 to FPB is considered low-intermediate. This outcome is expected as this biotype has not previously been subjected to selection with FPB. An increased RF would be verified after generations under selection pressure with the same herbicide. It is important to mention that the biotype survived at least 1× the recommended label rate at 25/20 °C and 4× at 35/30 °C, and the RF is highly influenced by the susceptibility level of the control biotype utilized for comparison. The ED_50_ obtained in our study is comparable to, in some cases exceeds, depending on the conditions, those reported for other FPB-resistant biotypes^29,30^ .

The resistance of *E. crus-galli* populations to FPB has been reported, and nontarget site resistance is the mechanism driving it.^27,28^ However, different genes have been reported as candidates for the FPB metabolism. Our RNA-seq analysis showed upregulation of genes from the *CYP*, *GST*, *UGT*, *ABC*, and *AKR* families, commonly associated with herbicide resistance. Even though several metabolism genes were upregulated in SAOJER-R, the *CYP* gene family is putatively involved in FPB metabolism, highlighting the three genes AH08.1281, BH09.2162, and CH01.638, annotated as *CYP71Z*-like, *CYP704C*-like, and *CYP81A*-like, respectively, because they were upregulated at both temperature conditions evaluated, considering that SAOJER-R is resistant at both temperature conditions but at different levels. They were also validated in qPCR analysis. Auxin-responsive *IAA*, *SAUR*, and *GH3* genes were not considered as candidates because their upregulation was not consistent across both temperature conditions tested. No *AFB* genes were differentially expressed in the resistant biotype. An *E. crus-galli* biotype from Hunan Province, China, showed two *CYP* (*CYP71C42* and *CYP71D55*), one *GST* (*GSTT2*), two *UGT* (*RhaT1* and *IAAGLU*), and two *ABC* (*ABCG1* and *ABCG25*) genes consistently upregulated in the FPB-resistant biotype.^29^ Some of these genes were found to be upregulated in at least one RNA-seq contrast in our study, such as *CYP71D55*, *CYP71C* subfamily members, and an *ABCG* subfamily member (Table S4). In addition, another biotype from China had the gene *CYP72A385* validated as conferring FPB resistance;^30^ however, this gene was not differentially expressed at any temperature condition in our study. Two *CYP81A*-family members, *CYP81A12* and *CYP81A21*, from *E. phyllopogon* were also validated to metabolize FPB.^43^ However, the *CYP71Z*-like gene identified in our study has not previously been associated with herbicide resistance. These results highlight the diversity of genes involved in the mechanism of resistance of the same species to the same herbicide, in this case, *E. crus-galli* resistant to FPB, which may be related to the high genetic diversity of this species.^13^

The biotype SAOJER-R is resistant to FPB and (1) has a resistance level highly influenced by temperature; (2) decreased amount of FPB and FPA in the plant when compared to the susceptible biotype; (3) effect of CYP450 inhibitor on reducing the RF and also increasing the amount of FPB and FPA detected in the plants; (4) consistent upregulation of *CYP* genes in both temperature conditions; and (5) consistent validation with qPCR. These five lines of evidence support the hypothesis of metabolic resistance to FPB, suggesting *CYP* genes as candidates, which corroborates other FPB resistance reports, where resistant *E. crus-galli* converts the FPB to florpyrauxifen-hydroxy acid by demethylation of aryl-methoxy groups, followed by a glucose conjugation, producing florpyrauxifen-hydroxy-glucose.^28^ In their study, the first metabolite was not present when the CYP450 inhibitor was applied, suggesting that the demethylation that produces the hydroxy acid metabolite is associated with CYP450 enzymes.^28^ In our studies, the biotype SAOJER-R showed auxinic symptoms and was highly injured in the first 7–14 days after FPB application, indicating that the herbicide is being recognized by its receptors, decreasing the target site mutation probability (but not completely ruled out). Auxinic herbicides are prone to metabolic degradation by CYP450 enzymes, considering also that 2,4-d resistance is associated with CYP450 metabolism in several species, such as *Amaranthus palmeri*, *Amaranthus tuberculatus*, *Conyza canadensis*, *C. sumatrensis*, and *Papaver rhoeas*.^44^ Our results also provide insights into the selectivity of FPB in rice, showing that metabolism might be the mechanism involved due to the low amount of FPB and FPA detected in LC–MS/MS analysis. However, because we did not quantify herbicide uptake, we cannot exclude reduced absorption as a causative factor. Previous studies have also shown that CYP450 inhibitor reduced the FPB ED_50_ from 217 to 90 g a.i. ha^–1^ in rice.^45^

Three *CYP* genes putatively involved in FPB resistance, *CYP71Z*-like, *CYP704C*-like, and *CYP81A*, were identified in SAOJER-R. The *CYP71Z*-like gene showed the highest differential expression when compared with the other two *CYP*s. It was 25 times more expressed in SAOJER-R than in VALE VERDE-S after FPB treatment. The overexpression of a gene from the *CYP71Z* subfamily, *CYP71Z2*, was reported to enhance rice resistance to the bacterial pathogen *Xanthomonas oryzae* *pv oryzae* by suppressing auxins in rice.^46^ Genes from the *CYP81A* family have been reported in several herbicide resistance studies,^7,19,43,47^ while *CYP704C1* was upregulated in *Raphanus raphanistrum* resistant to mesotrione^48^ and in *E. crus-galli* resistant to penoxsulam and FPB.^49^ The *CYP* gene family was also characterized as being involved in the resistance to other herbicides in different species. In *E. phyllopogon*, *CYP81A12* and *CYP81A21* drive the metabolism of penoxsulam, bensulfuron-methyl, ACCase inhibitors, and FPB.^7,17,43^ *CYP81A68* confers metabolic resistance to ALS and ACCase-inhibiting herbicides in *E. crus-galli*.^19^ A range of other *CYP81A* subfamily genes from *E. phyllopogon* were shown to metabolize many herbicides from different modes of action.^50^ In *Lolium rigidum*, *CYP81A10v7* confers metabolic resistance to herbicides diclofop-methyl, tralkoxydim, chlorsulfuron, mesotrione, atrazine, chlorotoluron, and trifluralin, which represent five different modes of action.^47^ According to these studies, the *CYP81A* family plays an important role in herbicide resistance; in the present study, one of our three main candidate genes is from *CYP81A*, but it was not the one with the highest level of expression. In addition, four *UGT* genes were also upregulated, BH01.674, BH03.2759, BH03.3274, and CH03.2963, which may be involved in the florpyrauxifen-hydroxy acid conjugation to glucose, which was a step previously identified in *E. crus-galli* biotypes from Arkansas and Missouri.^28^

The effect of temperature on FPB resistance level observed in SAOJER-R could be used to manage resistance during low-temperature periods. The lowest temperature tested in this study is still considered warm and decreased the SAOJER-R survival to 1× the FPB label rate, which suggests that resistance is diminished or reversed under lower temperatures. A similar temperature effect was observed for the same biotype SAOJER-R, which is metabolically resistant to cyhalofop-butyl, surviving up to 570 g a.i. ha^–1^ at 35/30 °C, but susceptible at the label rate of 285 g a.i. ha^–1^ when sprayed at 25/20 °C.^3^ The temperature response was also identified in other metabolism-based resistant weed species to 2,4-D,^51^ glyphosate,^11^ and pinoxaden.^52^ The effect of temperature on increasing the resistance level may be explained by two hypotheses: increased gene expression and/or increased enzymatic activity. The hypothesis of high temperature increasing gene expression was suggested for *Echinochloa colona* resistant to glyphosate associated with *AKR4*.^11^ The increased metabolism–enzymatic activity hypothesis at higher temperatures is supported by pinoxaden and fenoxaprop metabolic resistance in *Alopecurus myosuroides* associated with GST enzymes.^53–55^ Our expression data showed increased expression of *CYP* candidate genes at 35/30 °C; however, higher temperatures are likely to increase enzymatic activity as well. The effect of high temperatures in increasing herbicide resistance is a warning for future weed management if global temperatures continue to rise.^56^ Populations that currently exhibit susceptibility or metabolic resistance to herbicides will likely show reduced susceptibility or increased resistance, respectively, under continued global warming.

The resistance of *E. crus-galli* SAOJER-R biotype to FPB was characterized as metabolic-based. The resistant biotype metabolizes FPB and FPA faster than the susceptible biotype. The CYP450 inhibitor application decreased the RF and increased the amount of the herbicide in the resistant biotype. Utilizing different approaches (metabolic inhibitor, LC/MS–MS, RNA-seq, and qPCR validation), the candidate genes involved in FPB resistance were narrowed down. *CYP* genes are candidates for metabolizing FPB; however, further work is needed to functionally validate them. Additionally, other genes may contribute to the additive effects of the resistance. The mechanism of resistance was highly affected by temperature conditions with the resistance level decreasing at lower temperatures.

## Supplementary Material



## Data Availability

RNA-seq short-read data are available under BioProject PRJNA1076494 in GenBank (accessions SRR27966632–SRR27966661). All other data are provided in the manuscript or Supporting Information Additional data sets will be made available upon reasonable request.

## Notes

LC–MS/MS calibration curves and technical-grade standard retention times; comprehensive stability analysis of the housekeeping genes utilized in qPCR; auxin-responsive genes upregulated in the resistant compared to the susceptible biotype; auxin-responsive indole-3-acetic acid (*IAA*), auxin efflux carrier (*PIN*), and auxin-responsive factors (*ARF*) genes downregulated; qPCR validation of additional *CYP*, *GST*, *UGT*, and *GH3* genes upregulated in the RNA-seq; *CYP* phylogenetic tree utilizing 251 genes; karyotype of *E. crus-galli* showing the *CYP* gene locations; primers utilized in the qPCR validation; fresh biomass data of untreated and NBD-Cl-treated plants; fresh biomass data of untreated and malathion-treated plants; annotation of genes upregulated in the RNA-seq; and annotation of genes downregulated in the RNA-seq (PDF)

## ∥

Corteva Agriscience, Indianapolis, IN, USA

## Abbreviations used

(ALS): acetolactate synthase
(AKR): aldo-keto reductases
(ANOVA): analysis of variation
(ARF): auxin-responsive factors
(AFB): auxin receptor F-box
(ABC): ATP-binding cassette
(CYP and CYP450): cytochrome P450 monooxygenases
(DAT): days after treatment
(DEGs): differentially expressed genes
(ED_50_): effective dose that reduce 50% of the dry biomass or that provides 50% of control
(FPA): florpyrauxifen acid
(FPB): florpyrauxifen-benzyl
(GAPDH): glyceraldehyde-3-phosphate dehydrogenase
(GST): glutathione-*S*-transferase
(HAT): hours after treatment
(IAA): indole-3-acetic acid
(LFC): Log2Fold Change
(NBD-Cl): 4-chloro-7-nitrobenzofurazan
(PER): peroxidase
(ppm): parts per million
(RF): resistance factor
(RUB): rubisco
(UGT): UDP-glycosyltransferase
(28S): 28S rRNA.
