# Supplementary material for: Cytochrome P450 Enzymes Are Associated with the Innate Metabolic Resistance of Echinochloa crus-galli to Florpyrauxifen-Benzyl
Source: J Agric Food Chem. 2026 Oct 23;74(43):33645–61. doi: 10.1021/acs.jafc.6c06421 (PMC13741836; doi:10.1021/acs.jafc.6c06421)
Supplement: Supplementary file 1 [file jf6c06421_si_001.pdf]

SUPPORTING INFORMATION

Cytochrome P450 enzymes are associated with the innate metabolic resistance of *Echinochloa crus-galli* to florpyrauxifen-benzyl

Luan Cutti[a†], Eduardo C. Rudell[a], Crystal Sparks[b], Guilherme Menegol Turra[a], Paula Sinigaglia Angonese[a], Estéfani Sulzbach[a], Angela Da Cas Bundt[c], Catarine Markus[a], Franck E. Dayan[b], Todd A. Gaines[b], Aldo Merotto[a*]

[a] Crop Science Department, Federal University of Rio Grande do Sul, Porto Alegre, RS, Brazil, 91540-000.

[b] Department of Agricultural Biology, Colorado State University, Fort Collins, Colorado, USA, 80523.

[c] Corteva Agriscience, Barueri, SP, Brazil, 06460-000.

[†] Present address: Corteva Agriscience, Indianapolis, IN, USA.

*Corresponding author: aldo.merotto@ufrgs.br

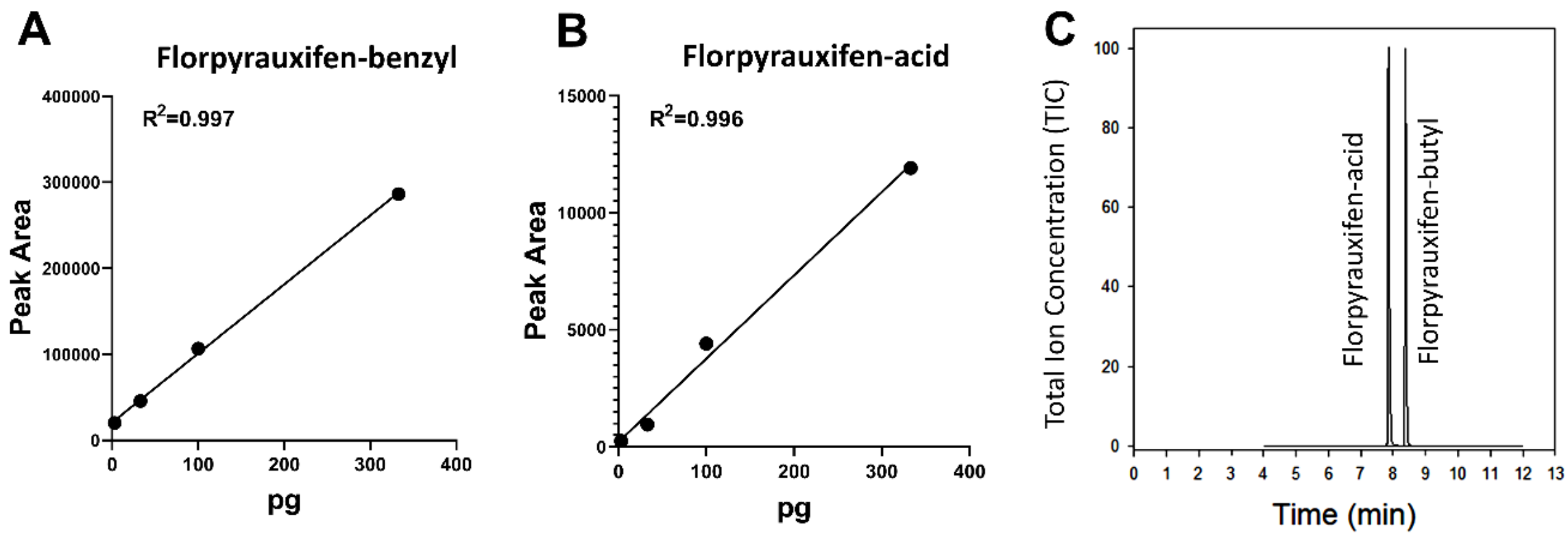

Figure S1. florpyrauxifen-benzyl (A) and florpyrauxifen acid (B) calibration curves in LC-MS/MS. (C) Technical-grade standard retention times.

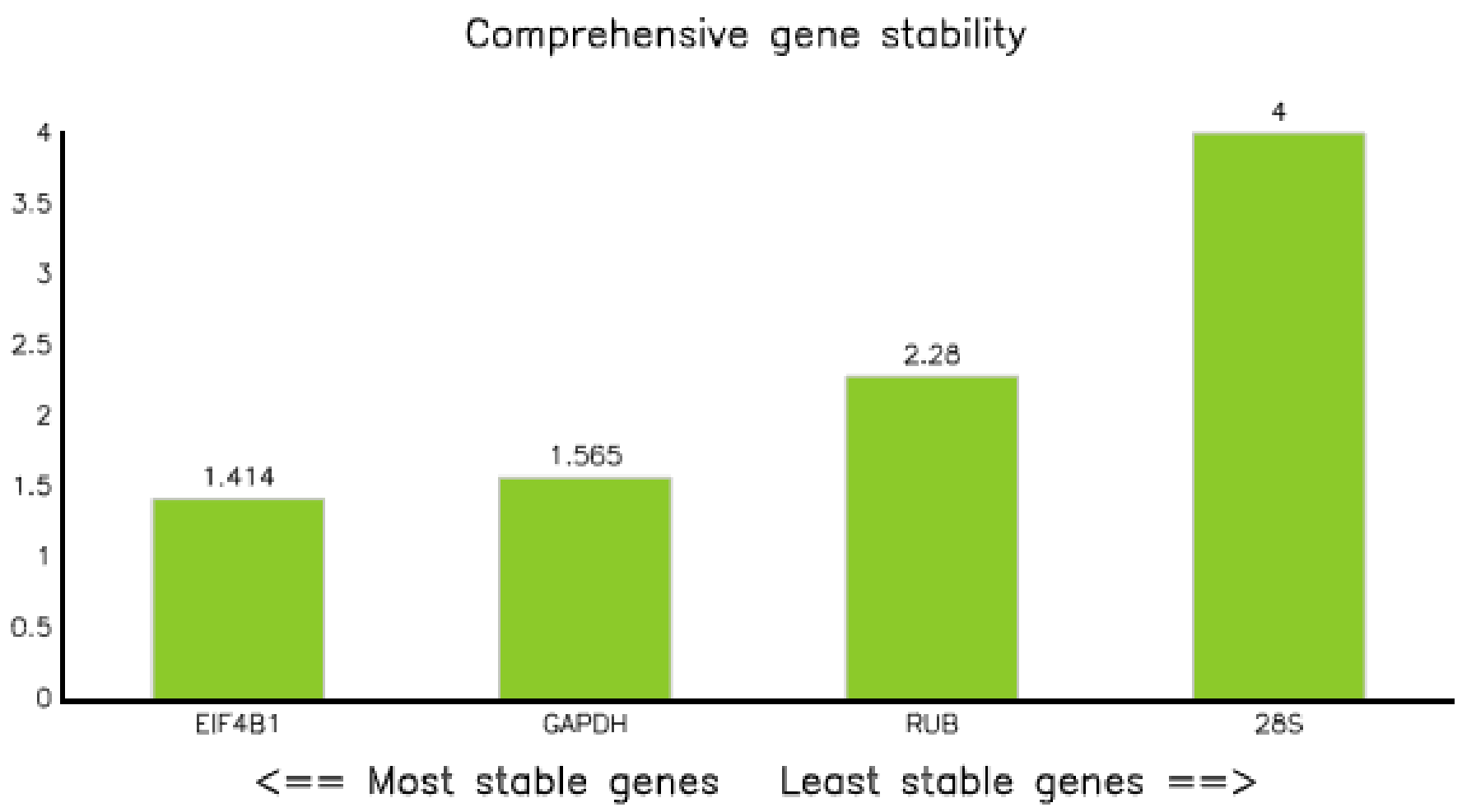

Figure S2. Comprehensive stability analysis of the housekeeping genes utilized in qPCR. The analysis was performed in RefFinder (https://www.ciidirsinaloa.com.mx/RefFinder-master/?type=reference), which integrates the currently available major computational programs (geNorm, Normfinder, BestKeeper, and the comparative Ct method) to compare and rank the tested candidate reference genes.

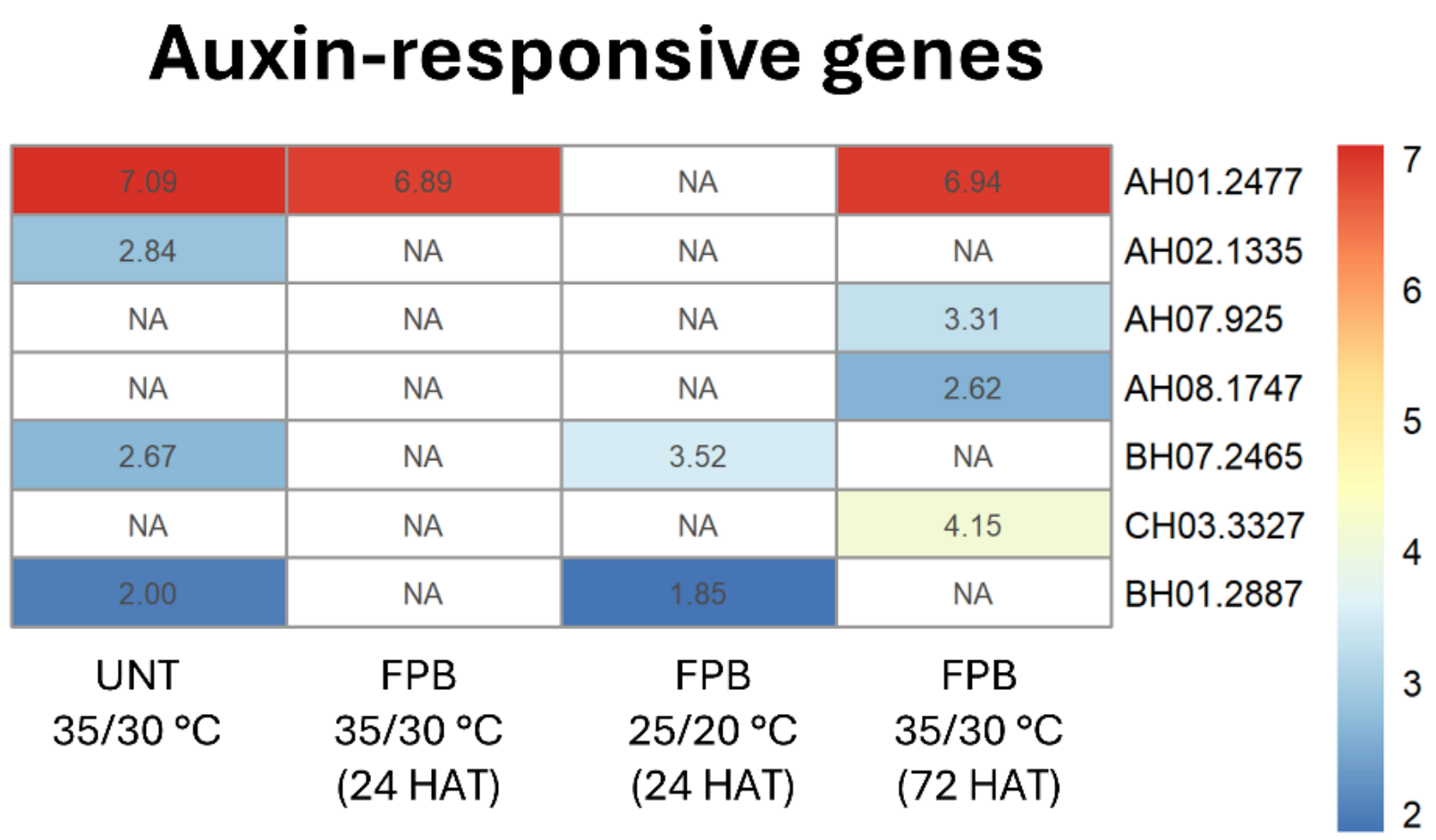

Figure S3. Auxin-responsive genes up-regulated in the resistant SAOJER-R compared to the susceptible VALE VERDE-S *Echinochloa crus-galli* biotype under four conditions (untreated (UNT) and treated with florpyrauxifen-benzyl (FPB)). “NA” indicates no differential expression in that condition.

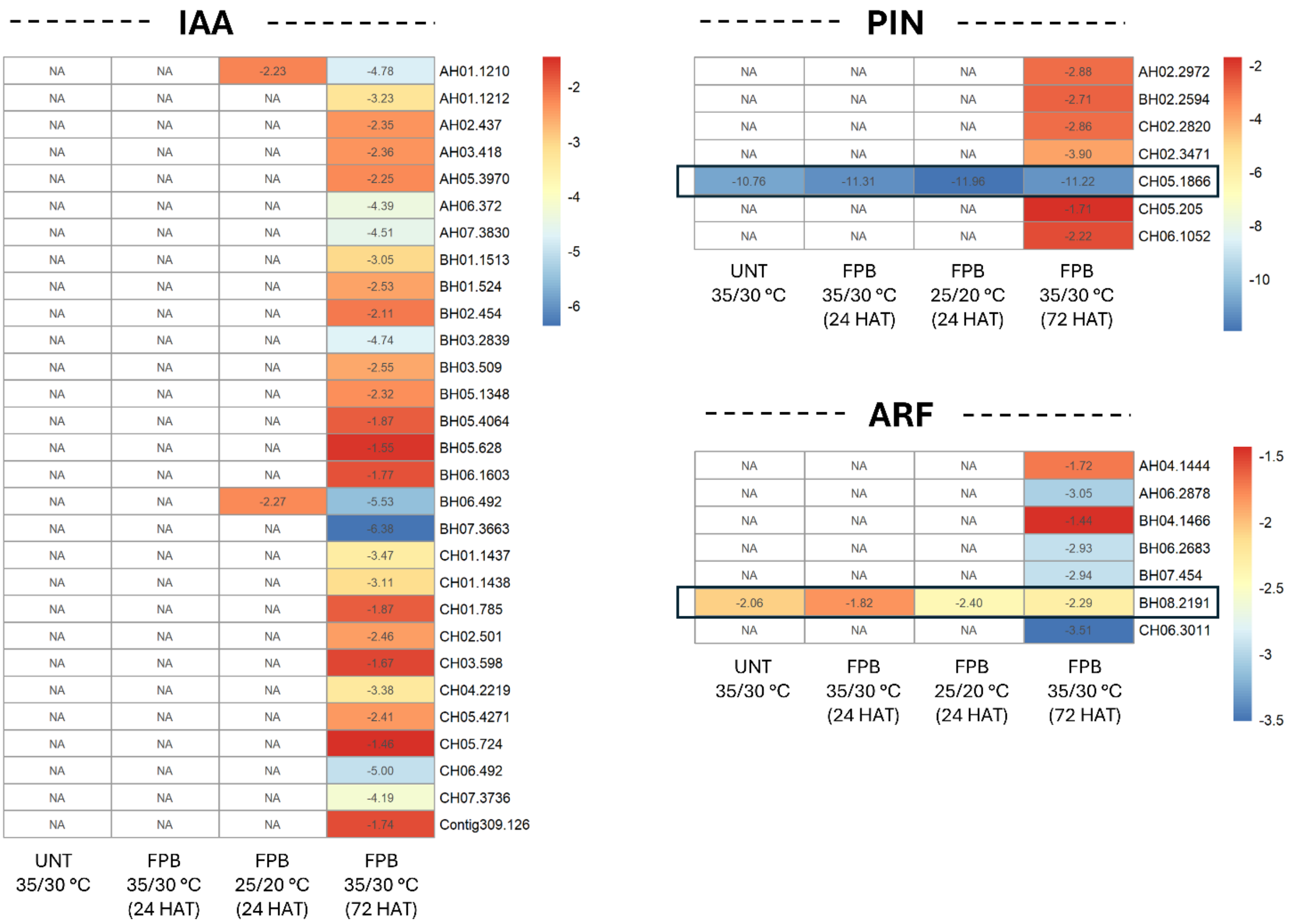

Figure S4. Auxin-responsive indole-3-acetic acid (*IAA*), auxin efflux carrier (*PIN*), and auxin-responsive factors (*ARF*) genes down-regulated in the resistant SAOJER-R compared to the susceptible VALE VERDE-S *Echinochloa crus-galli* biotype under four conditions (untreated (UNT) and treated with florpyrauxifen-benzyl (FPB)). The genes down-regulated in both contrasts "R vs. S sprayed 35/30 °C (24 HAT)" and "R vs. S sprayed 25/20 °C (24 HAT)" are highlighted. "NA" indicates no differential expression in that condition.

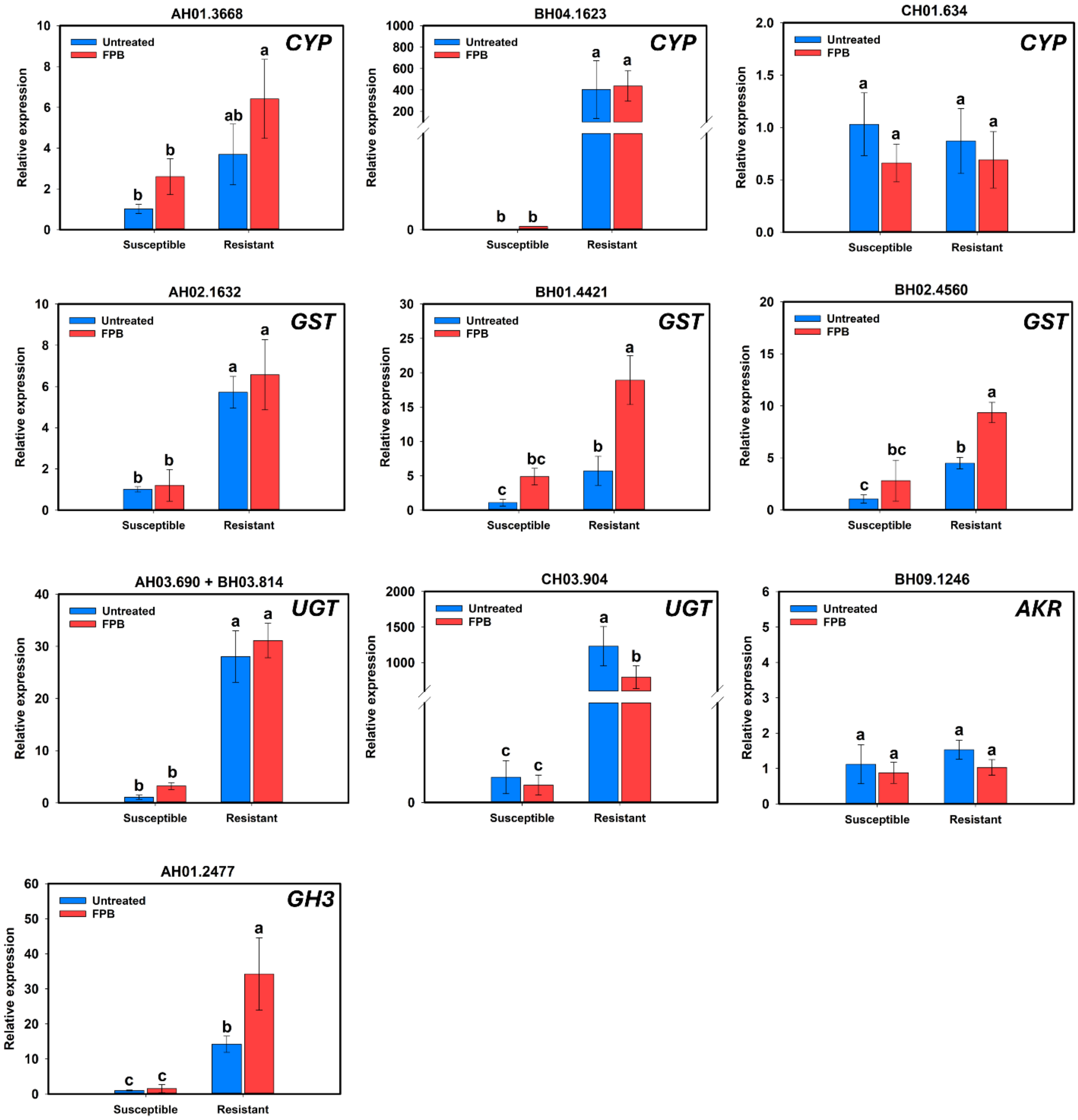

Figure S5. qPCR validation of additional *CYP*, *GST*, *UGT*, and *GH3* genes up-regulated in the RNA-seq analysis. Relative expression of selected genes from *CYP*, *GST*, *UGT*, and *GH3* families up-regulated only in one temperature condition in the RNA-seq, utilizing SAOJER-R and VALE VERDE-S *Echinochloa crus-galli* biotypes, untreated and treated with florpyrauxifen-benzyl (FPB), and collected at 24 HAT at 35/30 °C. Vertical bars indicate the confidence interval (95% interval).

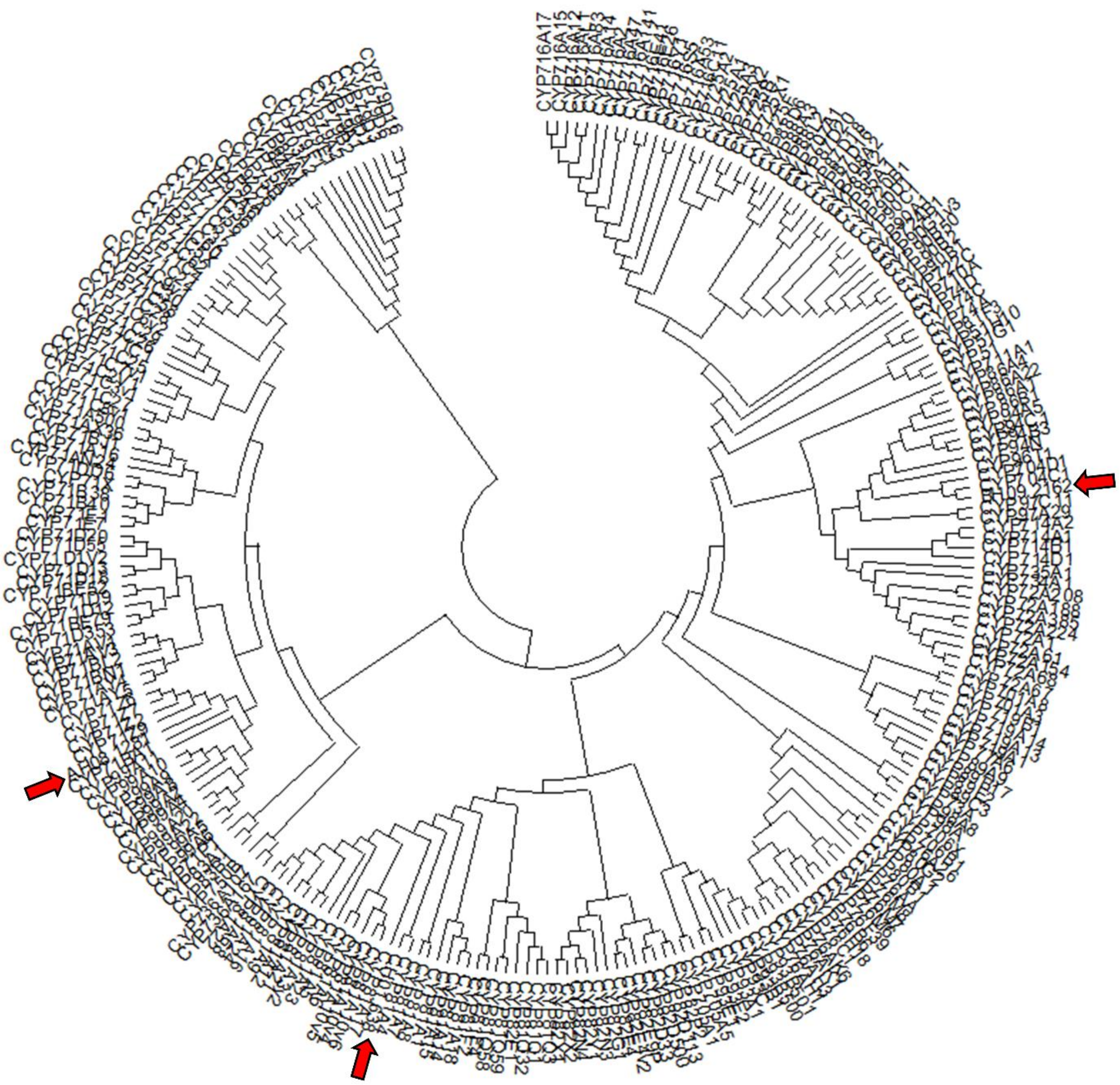

Figure S6. *CYP* genes phylogenetic tree utilizing 251 genes to annotate the candidate genes AH08.1281, BH09.2162, and CH01.638, indicated by the red arrows.

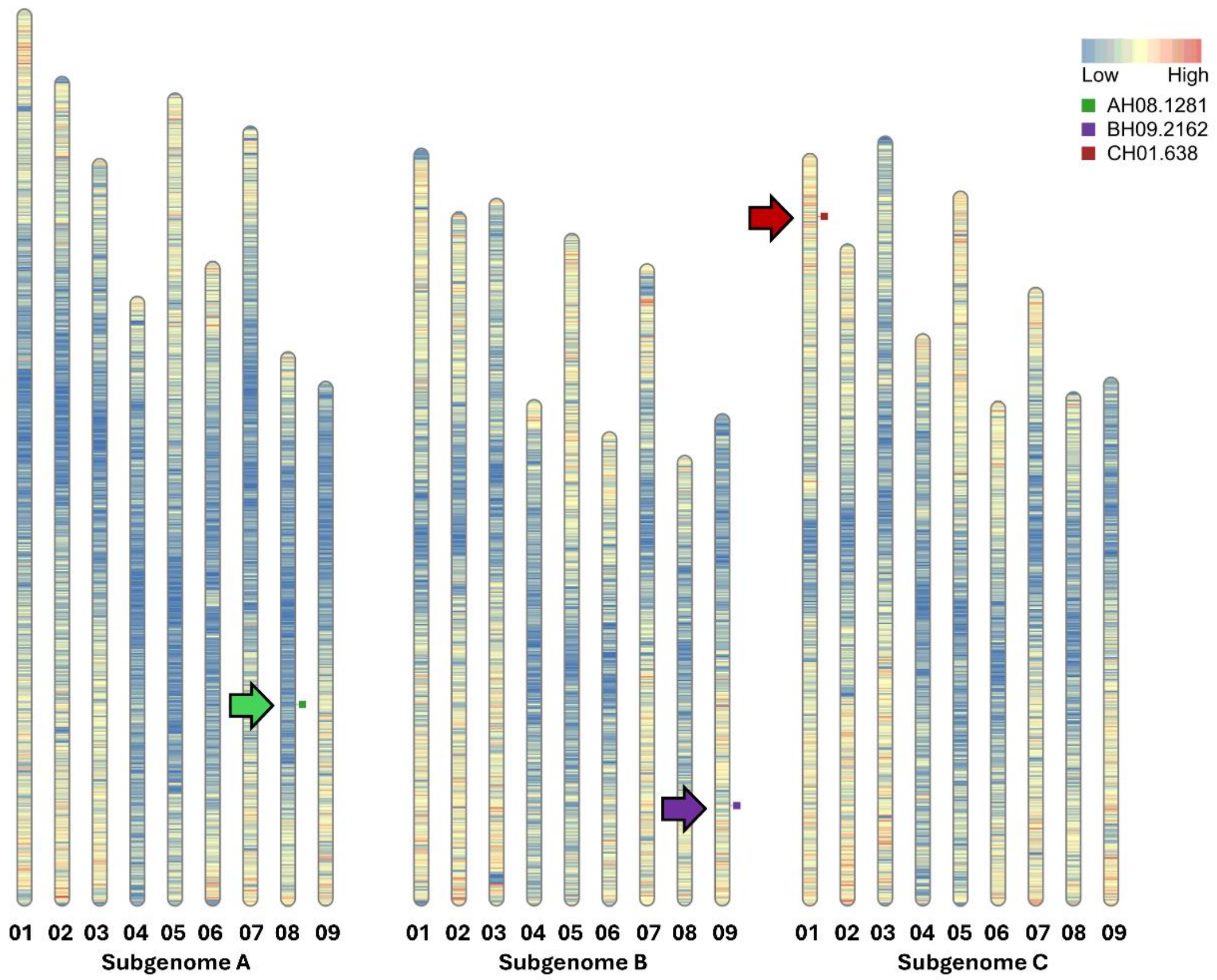

Figure S7. Karyotype of *Echinochloa crus-galli* showing the three *CYP* candidate gene locations.

Table S1. Primers utilized in the qPCR validation of selected genes up-regulated in the RNA-seq

Gene ID	Gene family	Sequence	Fragment size (bp)	Temperature
Housekeeping	*eIF4B1*	GGGAAGTGATTTTGCAGGAG<br>GAGGCTTGGTCAGAACCATC	190	64
AH01.3668	*CYP*	ATGCTGAAGGTGGACGAGAT<br>TTGGTGATGCTGTGGAACAT	80	64
AH08.1281	*CYP*	GCAAGGCTTCCATCTAGCAT<br>GCAAACATCCCTTCCCTGTA	117	64
BH09.2162	*CYP*	AGATGGGCAGAAACCATCAG<br>GTCCCGCAGGTACTTGTTGT	110	60
CH01.634	*CYP*	GATGGCGTTGAGGTTGACAT<br>GAGCTCCTGAAGCACACTGC	117	60
CH01.638	*CYP*	GCAGCCTATGTGGTGGCTAT<br>GCTCTTGCCATTGATTCCTC	99	60
BH04.1623	*CYP*	GGGGCCTAAGGAGATCAAGA<br>CGACGTGAACCATGGAGAG	83	60
AH02.1632	*GST*	TCGCTAGAAACCCATTCGGTG<br>GAGGATGTACCTCGCGATTG	87	60
BH01.4421	*GST*	TGCAGGACAAGATCAAGCTG<br>CTGATCAGGTCCAGGCTCTC	139	64
BH02.4558	*GST*	TTCTGTTCCCACGAAGCTCT<br>TCACTTCTGCTGCTGCAACT	123	64
BH02.4560	*GST*	CTTCGTGAAGGAGACGAAGG<br>TGAGCCAACACACTGAGGAC	117	64
BH05.515	*GST*	ATCGCTCTCAACCTCAAAGG<br>CCATCCACAAAAGCTGGAAT	113	60
BH05.3318	*GST*	GTGCAATGTTCAAGGCAGAA<br>ACTTCGGCTTGTCCTTCAGA	84	60
CH01.480 +<br>BH03.3871	*GST*	AGCTCGGGGAGAAGGAGTT<br>GCTCGTACGTGAGGAACCAC	93	60
AH03.690 and<br>BH03.814	*UGT*	TACGTGAGCTTTGGGAGCTT<br>GCCATCTGGTAGCCGTACAT	147	60
BH01.674	*UGT*	GAGGAGACGATCAGGTCCAA<br>GCCGAGAAAGTGGAGAACAA	119	60
BH03.2759 +<br>CH03.2963	*UGT*	TGGTCTGATCAACCCATGAA<br>GCCATCCATCACCTCTCCTA	132	64
BH03.3274	*UGT*	CAGTACGGCCAGCCTATGAT<br>ATCCTTGTGCACCCTCACTC	102	60
CH03.904	*UGT*	ATGAGACAGAGGGCCAAGAA<br>CATTATGCGTTTCAGGAGCA	102	64
AH02.477	*ABC*	GATGGTGTCGACATTTGCAC<br>CCTCGAACATGGTTGGTTCT	85	60
BH08.936	*ABC*	CGTTCTGTGACCACGAGCTA<br>GGGTCTCCAGCACTGTAAGC	91	60

CH07.703	*ABC*	TTTCAAATCGCTTTGGTTCC<br>AAATCGACGGAAGCTCTGAA	139	60
AH09.542	*AKR*	AAAGGATGCACACCATCACA<br>CAGATAATGCCCCCACATTC	121	60
BH05.2320	*AKR*	AAAGCTCGTGCTATCGGTGT<br>GTTTTCGGAGCTTGTCTTGC	133	64
BH09.1246	*AKR*	ATAATGCCCATCGACCTGAG<br>GGATTTCAGCAATGGCAAGT	133	60
CH09.664	*AKR*	ACCAAGGTTTCAGCCAGAGA<br>GACATCGCTTCCCTGATGAT	133	60
AH01.2685	*PER*	GATAGCAAAAACGCCGACAT<br>CCCGAACTTCTTCTCGAGTG	84	60
AH01.2477	*GH3*	AAGTGCTCCAGCGACGAGT<br>GCCTTATCGGTCTTGTCGAA	81	60

Table S2. Fresh biomass data of untreated and NBD-Cl-treated (270 g ha^{-1}) plants compared by *t*-test

Biotype	Inhibitor	Herbicide dose	Rep	Fresh biomass (g)	Average	*t*-test
VALE VERDE	No inhibitor	Untreated	1	58.815	61.247	0.004 (*)
VALE VERDE	No inhibitor	Untreated	2	62.350		
VALE VERDE	No inhibitor	Untreated	3	68.124		
VALE VERDE	No inhibitor	Untreated	4	70.123		
VALE VERDE	No inhibitor	Untreated	5	46.824		
VALE VERDE	NBD-Cl	Untreated	1	46.565	42.815	
VALE VERDE	NBD-Cl	Untreated	2	48.989		
VALE VERDE	NBD-Cl	Untreated	3	43.255		
VALE VERDE	NBD-Cl	Untreated	4	35.844		
VALE VERDE	NBD-Cl	Untreated	5	39.420		
SAOJER-R	No inhibitor	Untreated	1	41.048	49.318	0.595 (ns)
SAOJER-R	No inhibitor	Untreated	2	51.201		
SAOJER-R	No inhibitor	Untreated	3	56.222		
SAOJER-R	No inhibitor	Untreated	4	45.028		
SAOJER-R	No inhibitor	Untreated	5	53.093		
SAOJER-R	NBD-Cl	Untreated	1	52.147	47.016	
SAOJER-R	NBD-Cl	Untreated	2	49.323		
SAOJER-R	NBD-Cl	Untreated	3	46.339		
SAOJER-R	NBD-Cl	Untreated	4	35.256		
SAOJER-R	NBD-Cl	Untreated	5	52.013		

Asterisk (*) = significant ($p \leq 0.05$); ns = non-significant.

Table S3. Fresh biomass data of untreated and malathion-treated (1,500 g ha^{-1}) plants compared by *t*-test

Biotype	Inhibitor	Herbicide dose	Rep	Fresh biomass (g)	Average	*t*-test
VALE VERDE	No inhibitor	Untreated	1	58.815	61.247	0.509 (ns)
VALE VERDE	No inhibitor	Untreated	2	62.350		
VALE VERDE	No inhibitor	Untreated	3	68.124		
VALE VERDE	No inhibitor	Untreated	4	70.123		
VALE VERDE	No inhibitor	Untreated	5	46.824		
VALE VERDE	Malathion	Untreated	1	61.400	58.074	
VALE VERDE	Malathion	Untreated	2	64.167		
VALE VERDE	Malathion	Untreated	3	53.680		
VALE VERDE	Malathion	Untreated	4	56.525		
VALE VERDE	Malathion	Untreated	5	54.600		
SAOJER-R	No inhibitor	Untreated	1	41.048	49.318	0.353 (ns)
SAOJER-R	No inhibitor	Untreated	2	51.201		
SAOJER-R	No inhibitor	Untreated	3	56.222		
SAOJER-R	No inhibitor	Untreated	4	45.028		
SAOJER-R	No inhibitor	Untreated	5	53.093		
SAOJER-R	Malathion	Untreated	1	48.072	53.796	
SAOJER-R	Malathion	Untreated	2	67.313		
SAOJER-R	Malathion	Untreated	3	54.136		
SAOJER-R	Malathion	Untreated	4	47.190		
SAOJER-R	Malathion	Untreated	5	52.267		

Asterisk (*) = significant ($p \leq 0.05$); ns = non-significant.

Table S4. Annotation of RNA-seq up-regulated genes from the *CYP, GST, UGT, ABC, AKR,* and *PER* families and auxin-responsive genes

Gene Family	Gene_ID	Functional annotation	Annotation source
CYP	BH09.2162	*CYP704C-like*	Phylogenetic tree
CYP	CH08.2063	*CYP707A7*	Swiss-Prot
CYP	AH03.3824	*CYP709B2*	Swiss-Prot
CYP	CH02.1797	*CYP709B2*	Swiss-Prot
CYP	AH09.105	*CYP71BE54*	Swiss-Prot
CYP	BH04.2296	*CYP71C1*	Swiss-Prot
CYP	AH08.34	*CYP71C2*	Swiss-Prot
CYP	BH04.2297	*CYP71C2*	Swiss-Prot
CYP	Contig398.30	*CYP71C2*	Swiss-Prot
CYP	AH01.3668	*CYP71D55*	Swiss-Prot
CYP	AH08.1281	*CYP71Z-like*	Phylogenetic tree
CYP	AH03.856	*CYP71E1*	Swiss-Prot
CYP	BH05.3776	*CYP71E1*	Swiss-Prot
CYP	CH04.2450	*CYP71P1*	Swiss-Prot
CYP	AH01.4462	*CYP74A2*	Swiss-Prot
CYP	Contig192.104	*CYP74A2*	Swiss-Prot
CYP	CH03.4074	*CYP78A5*	Swiss-Prot
CYP	BH01.1697	*CYP79A1*	Swiss-Prot
CYP	BH01.655	*CYP81A-like*	Swiss-Prot
CYP	CH01.634	*CYP81A-like*	Swiss-Prot
CYP	CH01.636	*CYP81A-like*	Swiss-Prot
CYP	CH01.638	*CYP81A-like*	Phylogenetic tree
CYP	BH07.2078	*CYP86B1*	Swiss-Prot
CYP	CH02.2199	*CYP87A3*	Swiss-Prot
CYP	BH04.1623	*CYP89A9*	Swiss-Prot
CYP	AH02.3222	*CYP76M5-like*	Swiss-Prot
GST	AH02.1632	*GST4*	Swiss-Prot
GST	CH02.1909	*GST4*	Swiss-Prot
GST	CH05.1180	*GST4*	Swiss-Prot
GST	BH02.4560	*GST23*	Swiss-Prot
GST	AH02.1628	*GSTF1*	Swiss-Prot
GST	CH02.2038	*GSTF1*	Swiss-Prot
GST	AH01.3038	*GSTF11*	Swiss-Prot
GST	BH01.3381	*GSTF11*	Swiss-Prot
GST	CH01.3622	*GSTF11*	Swiss-Prot
GST	AH01.5220	*GSTF8*	Swiss-Prot
GST	BH01.4990	*GSTF8*	Swiss-Prot
GST	CH01.5594	*GSTF8*	Swiss-Prot
GST	CH01.5595	*GSTF8*	Swiss-Prot
GST	BH01.3461	*GSTU6*	Swiss-Prot
GST	BH05.3318	*GSTT3*	Swiss-Prot
GST	CH01.480	*GSTU1*	Swiss-Prot

GST	BH05.515	*GSTZ1*	Swiss-Prot
GST	AH03.4158	*GSTZ5* (Protein IN2-1 homolog B)	Swiss-Prot
GST	BH02.4558	*HSP26-A* (*GSTX6*)	Swiss-Prot
GST	BH01.4421	*GSTZ5*	Swiss-Prot
UGT	AH03.690	*BX9* (DIMBOA UDP-glucosyltransferase BX9)	Swiss-Prot
UGT	BH03.814	*BX9* (DIMBOA UDP-glucosyltransferase BX9)	Swiss-Prot
UGT	AH03.2618	*UGT13248*	Swiss-Prot
UGT	AH09.252	*UGT13248*	Swiss-Prot
UGT	BH03.2759	*UGT13248*	Swiss-Prot
UGT	CH03.2963	*UGT13248*	Swiss-Prot
UGT	CH03.904	*UGT76F1*	Swiss-Prot
UGT	BH01.674	*UGT83A1*	Swiss-Prot
UGT	BH09.1289	*UGT85A24*	Swiss-Prot
UGT	CH09.1399	*UGT85A24*	Swiss-Prot
UGT	BH03.3274	*UGT91B1*	Swiss-Prot
AKR	AH09.542	*AKR2*	Swiss-Prot
AKR	CH09.664	*AKR3*	Swiss-Prot
AKR	BH05.2320	*AKR4C10*	Swiss-Prot
AKR	BH07.4194	*AKR4C9*	Swiss-Prot
AKR	BH09.1246	*DMAS1-D* (from *AKR* family)	Swiss-Prot
ABC	AH02.477	*ABCC3*	Swiss-Prot
ABC	AH06.326	*ABCC10*	Swiss-Prot
ABC	CH06.2054	*ABCC8*	Swiss-Prot
ABC	CH07.703	*ABCA8*	Swiss-Prot
ABC	BH08.936	*STR* (*ABCG*)	Swiss-Prot
PER	CH01.4139	*PER2*	Swiss-Prot
PER	CH02.1565	*PER2*	Swiss-Prot
PER	CH04.1232	*PER2*	Swiss-Prot
PER	AH01.2684	*PER15*	Swiss-Prot
PER	AH01.2685	*PER15*	Swiss-Prot
PER	AH06.1937	*PER52*	Swiss-Prot
PER	BH01.1726	*PER56*	Swiss-Prot
GH3	AH01.2477	*GH3.2*	Swiss-Prot
IAA	AH02.1335	*IAA4*	Swiss-Prot
IAA	AH07.925	*IAA7*	Swiss-Prot
SAUR	AH08.1747	*SAUR76*	Swiss-Prot
SAUR	BH07.2465	*SAUR32*	Swiss-Prot
SAUR	CH03.3327	*SAUR39*	Swiss-Prot
LAX	BH01.2887	*LAX3*	Swiss-Prot

Table S5. Annotation of RNA-seq down-regulated genes from the *IAA, PIN,* and *ARF* gene families

Gene Family	Gene_ID	Functional annotation	Annotation source
IAA	AH01.1210	*IAA30*	Swiss-prot
IAA	AH01.1212	*IAA12*	Swiss-prot
IAA	AH02.437	*IAA1-like*	Swiss-prot
IAA	AH03.418	*IAA24-like*	Swiss-prot
IAA	AH05.3970	*IAA31-like*	Swiss-prot
IAA	AH06.372	*IAA20-like*	Swiss-prot
IAA	AH07.3830	*IAA9-like*	Swiss-prot
IAA	BH01.1513	*IAA30*	Swiss-prot
IAA	BH01.524	*IAA14-like*	Swiss-prot
IAA	BH02.454	*IAA1*	Swiss-prot
IAA	BH03.2839	*IAA26*	Swiss-prot
IAA	BH03.509	*IAA24-like*	Swiss-prot
IAA	BH05.1348	*IAA1*	Swiss-prot
IAA	BH05.4064	*IAA31-like*	Swiss-prot
IAA	BH05.628	*IAA17-like*	Swiss-prot
IAA	BH06.1603	*IAA21*	Swiss-prot
IAA	BH06.492	*IAA20-like*	Swiss-prot
IAA	BH07.3663	*IAA9-like*	Swiss-prot
IAA	CH01.1437	*IAA12*	Swiss-prot
IAA	CH01.1438	*IAA30*	Swiss-prot
IAA	CH01.785	*IAA13-like*	Swiss-prot
IAA	CH02.501	*IAA1*	Swiss-prot
IAA	CH03.598	*IAA24-like*	Swiss-prot
IAA	CH04.2219	*IAA12*	Swiss-prot
IAA	CH05.4271	*IAA31-like*	Swiss-prot
IAA	CH05.724	*IAA17-like*	Swiss-prot
IAA	CH06.492	*IAA20-like*	Swiss-prot
IAA	CH07.3736	*IAA9-like*	Swiss-prot
IAA	Contig309.126	*IAA13-like*	Swiss-prot
PIN	AH02.2972	*probable auxin efflux carrier component 8*	Swiss-prot
PIN	BH02.2594	*auxin efflux carrier component 3a*	Swiss-prot
PIN	CH02.2820	*auxin efflux carrier component 3a*	Swiss-prot
PIN	CH02.3471	*probable auxin efflux carrier component 8*	Swiss-prot
PIN	CH05.1866	*probable auxin efflux carrier component 3b*	Swiss-prot
PIN	CH05.205	*probable auxin efflux carrier component 1b*	Swiss-prot
PIN	CH06.1052	*probable auxin efflux carrier component 1c*	Swiss-prot
ARF	AH04.1444	*auxin response factor 23-like*	Swiss-prot
ARF	AH06.2878	*auxin response factor 19-like*	Swiss-prot
ARF	BH04.1466	*auxin response factor 23-like*	Swiss-prot
ARF	BH06.2683	*auxin response factor 19-like*	Swiss-prot
ARF	BH07.454	*auxin response factor 5-like*	Swiss-prot
ARF	BH08.2191	*auxin response factor 21-like*	Swiss-prot
ARF	CH06.3011	*auxin response factor 19-like*	Swiss-prot
